# A fast strapdown gyrocompassing algorithm based on INS differential errors

**DOI:** 10.1038/s41598-023-42235-6

**Published:** 2023-09-15

**Authors:** M. A. Amiri Atashgah, Hamed Mohammadkarimi, Mehrdad Ebrahimi

**Affiliations:** 1 Faculty of New Sciences and Technologies, University of Tehran, Tehran, Iran; 2 Department of Aerospace Engineering, Amirkabir University of Technology, Tehran, Iran

**Keywords:** Aerospace engineering, Electrical and electronic engineering

## Abstract

This paper presents an enhanced algorithm for inertial gyrocompassing using strapdown sensors, which performs faster than the other available ones. The proposed algorithm is based on differential errors in an inertial navigation system and requires only the output of the inertial measurement unit while extracting and compensating for the inertial sensor errors. After eliminating the error of the inertial sensors, which is accomplished swiftly, the coarse alignment algorithm performs with error-compensated sensors, and the true north is extracted accurately. The number of non-observable parameters of the algorithm is equal to that of the fine alignment algorithm; therefore, its accuracy is the same as that of a well-tuned fine alignment. Numerical simulations and lab experiments demonstrate that the proposed method performs heading estimation in the time required to perform the coarse alignment, which is faster than the existing fine alignment algorithms.

## Introduction

Flying vehicles usually use ‘initial alignment’ as the first step of navigation^1^; this means that they correct their directions at the beginning of a flight. Alignment means the relationship between the Body (B) and the Navigation (N) coordinate system^2^. The word ‘leveling’ refers to calculating the angles of roll and pitch, which are the vehicle deviation from the horizon. The meaning of ‘gyrocompassing’ is finding the direction of the geographic north.

Inaccurate initial alignment causes imprecise navigation. Thus, achieving a high degree of alignment over a short time is necessary. If the initial alignment is accomplished in a stationary mode, the level angles (ϕ and θ) extraction is performed by the accelerometers; Thus, the trouble of the alignment procedure will be gyrocompassing (north-finding). Usually, there is a limited time to calculate the heading angle, however most gyrocompassing algorithms are time-consuming. Therefore, it is necessary to access a gyrocompassing algorithm with a short time and high accuracy.

Various alignment techniques can be divided into inertial, non-inertial, and hybrid methods. The inertial methods use inertial sensors to generate the transformation matrix from the body to the navigation coordinate system and work based on gravity and Earth rate measurements. Non-inertial methods are based on other physical properties; For example, the magnetic compass utilizes the Earth’s magnetic field and finds the magnetic north instead of the true north. Hybrid methods integrate the information from the inertial and non-inertial methods and have the advantage of these two categories simultaneously.

In inertial alignment, the goal is to determine the level and heading angles by strapdown inertial sensors, including three gyros and three accelerometers, and use an algorithm implemented in the processor. The alignment algorithm estimates the navigation system errors (including inertial sensor, numerical computation, and initial condition errors) and corrects the navigation solution.

In general, it can be stated that inertial alignment is categorized as ‘stationary alignment’ and ‘in-motion alignment’. Stationary alignment (the subject of this study) is completed in two successive phases, and accuracy is improved at each phase. These steps are ‘Coarse alignment’^3^ and ‘Fine alignment’^4^. The first phase (coarse alignment) provides a rough estimate of initial attitudes. In the second phase (fine alignment), a filter (regularly Kaman filters/KF) is used to refine the alignment and estimate inertial sensor errors before flight^5^. In this paper we have proposed a new algorithm which combines the advantages of the two conventional alignment algorithms (i.e., coarse and fine). The new algorithm is as fast as the coarse alignment and as accurate as the fine alignment. In other words, the new alignment algorithm is fast and accurate.

Reference^6^ proposes an algorithm for the stationary alignment of rocket navigation systems. A filter is utilized to decrease the noise in the inertial measurement. Reference^7^ proposes an approach based on the expansion of the measurements, where the sensors’ biases are estimated more quickly and accurately. Despite the effectiveness of the proposed approach, it could not improve the convergence rate of the platform misalignments; since it depends directly on the unobservable uncompensated biases, which the Kalman filter cannot estimate.

In Ref.^8^, a new method is proposed for the initial alignment. A Kalman Filter is applied for the fine alignment, while the horizontal acceleration of gravity is also taken into the measurement equations. This approach enhances the tracking ability of the KF for the attitude angle’s change. In Ref.^9^, a multi-rate self-alignment algorithm enhances the KF capability in estimating biases. In Ref.^10^, the design principle of a dual-axis rotating SINS is proposed to improve the slow convergence rate and the low accuracy during the initial alignment and self-calibration.

Reference^11^ presents a SINS error analysis of coarse alignment formulations. The proposed formulation does not imply normality and orthogonality errors. In Ref.^12^ general expression for the SINS coarse alignment errors is derived, which is valid regardless of the inertial measurement unit (IMU) orientation.

The fine alignment problem has been studied in many references, such as^13–19^. Reference^20^ provides a quick method for accurate alignment; in this method, the roll and pitch rates are used to estimate the heading angle. In Ref.^21^, the navigation block is triggered with a particular mechanical motion, increasing precision and reducing fine alignment time. In Ref.^22^, the ‘multi-position’ technique is described in fine alignment; In the proposed method, the attitude of the IMU carrier is changed, and consequently, the observability of the inertial navigation system is improved. This technique reduces the alignment error.

A free IMU can perform fine alignment algorithms in a stationary mode without external aids. ‘Self-alignment’ is a process that is independent of external aiding navigation systems^23^. The advantage of this method is the independence of the INS from any external data; the disadvantages are as follows: some parameters become non-observable, sensitivity to noise of the inertial sensors is high, and the time required for the convergence of the algorithm is about 5–10 min.

As mentioned, the coarse alignment algorithm is fast and imprecise, while the fine alignment algorithm is slow and accurate. The algorithm presented in this paper is fast as the coarse alignment and accurate as the fine alignment. In other words, the proposed algorithm has the advantages of both traditional algorithms.

The organization of this paper is as follows: in “Rotation of small angles”, the relation between the small rotation angles and the Euler angles is derived. In “The proposed algorithm formulation”, the mathematical formulation of the new algorithm is presented. In “Methods”, the proposed algorithm is simulated and verified numerically, and lastly, the conclusion is made in “Simulation and experiments”.

## Rotation of small angles

The following relation holds between two frames when the rotation angles are small^24^:

1 $$ {\mathbf{R}}^{{{\hat{\text{N}}\text{N}}}} = {\mathbf{E}} + {\upvarepsilon }{\mathbf{R}}^{{{\hat{\text{N}}\text{N}}}} $$

In the above equation, ${\mathbf{R}}^{{{\hat{\text{N}}\text{N}}}}$ is the rotation tensor of the estimated navigation frame (${\hat{\text{N}}}$) relative to the true navigation frame (N); **E** is the identity tensor and ${\upvarepsilon }{\mathbf{R}}^{{{\hat{\text{N}}\text{N}}}}$ is the skew-symmetric form of the perturbation tensor (${\upvarepsilon }{\mathbf{r}}^{{{\hat{\text{N}}\text{N}}}}$), which is modeled as follows:

2 $$ [{\upvarepsilon }{\mathbf{r}}^{{{\hat{\text{N}}\text{N}}}} ]^{{{\hat{\text{N}}}}} = [{\upvarepsilon }{\mathbf{r}}^{{{\hat{\text{N}}\text{N}}}} ]^{{\text{N}}} = [\begin{array}{*{20}c} {{\upvarepsilon }\varphi } & {{\upvarepsilon }\theta } & {{\upvarepsilon }\psi } \\ \end{array} ]^{{\text{T}}} \to [{\upvarepsilon }{\mathbf{R}}^{{{\hat{\text{N}}\text{N}}}} ]^{{\text{N}}} = \left[ {\begin{array}{*{20}c} 0 & { - \varepsilon \psi } & {\varepsilon \theta } \\ {\varepsilon \psi } & 0 & { - \varepsilon \varphi } \\ { - \varepsilon \theta } & {\varepsilon \varphi } & 0 \\ \end{array} } \right] $$

where ${\upvarepsilon }\varphi$, ${\upvarepsilon }\theta$ and ${\upvarepsilon }\psi$ represent three small rotation angles. According to Ref.^24^, $[{\mathbf{R}}^{{{\hat{\text{N}}\text{N}}}} ]^{{\text{N}}} = [\overline{{\text{T}}} ]^{{{\hat{\text{N}}\text{N}}}}$ where $[{\text{T}}]^{{{\hat{\text{N}}\text{N}}}}$ is the transformation matrix of the N frame to the ${\hat{\text{N}}}$ frame; Utilizing this property, Eq. (1) can be expressed in the navigation frame:

3 $$ [\varepsilon {\mathbf{R}}^{{\widehat{{\text{N}}}{\text{N}}}} ]^{{\text{N}}} = [{\mathbf{R}}^{{\widehat{{\text{N}}}{\text{N}}}} ]^{{\text{N}}} - [{\mathbf{E}}]^{{\text{N}}} = [\overline{{\text{T}}} ]^{{\widehat{{\text{N}}}{\text{N}}}} - [{\mathbf{E}}]^{{\text{N}}} = [{\text{T}}]^{{{\text{N}}\widehat{{\text{N}}}}} - [{\mathbf{E}}]^{{\text{N}}} = [{\text{T}}]^{{{\text{NB}}}} [{\text{T}}]^{{\widehat{{{\text{BN}}}}}} - [{\mathbf{E}}]^{{\text{N}}} $$

Transposing Eq. (3) results in:

4 $$ - [{\upvarepsilon }{\mathbf{R}}^{{{\hat{\text{N}}\text{N}}}} ]^{{\text{N}}} = [{\overline{\text{T}}}]^{{{{\text{B}\hat{\text{N}}}}}} [{\overline{\text{T}}}]^{{{\text{NB}}}} - [{\mathbf{E}}]^{{\text{N}}} \,\,\,\,\,\, \to \,\,\,\,[{\upvarepsilon }{\mathbf{R}}^{{{\hat{\text{N}}\text{N}}}} ]^{{\text{N}}} = [{\mathbf{E}}]^{{\text{N}}} - [{\overline{\text{T}}}]^{{{{\text{B}\hat{\text{N}}}}}} [{\text{T}}]^{{{\text{BN}}}} $$

Now suppose that $$[{\text{T}}]^{{{\text{BN}}}} = f(\overset{\lower0.5em\hbox{$\smash{\scriptscriptstyle\frown}$}}{\psi } ,\overset{\lower0.5em\hbox{$\smash{\scriptscriptstyle\frown}$}}{\theta } ,\overset{\lower0.5em\hbox{$\smash{\scriptscriptstyle\frown}$}}{\varphi } )$$ and $$[{\text{T}}]^{{{{\text{B}\hat{\text{N}}}}}} = f(\hat{\psi },\hat{\theta },\hat{\varphi })$$; where $$\overset{\lower0.5em\hbox{$\smash{\scriptscriptstyle\frown}$}}{\psi } ,\,\overset{\lower0.5em\hbox{$\smash{\scriptscriptstyle\frown}$}}{\theta } ,\,\overset{\lower0.5em\hbox{$\smash{\scriptscriptstyle\frown}$}}{\varphi }$$ are true and $$\hat{\psi },\,\hat{\theta },\,\hat{\varphi }$$ are estimated Euler angles and ‘*f*’ is a matrix function that transforms navigation to body frame and is defined as follows^24^:

5 $$ f(\psi ,\theta ,\varphi ) = \left[ {\begin{array}{*{20}c} {\cos \psi \cos \theta } & {\sin \psi \cos \theta } & { - \sin \theta } \\ {\cos \psi \sin \theta \sin \varphi - \sin \psi \cos \phi } & {\sin \psi \sin \theta \sin \varphi + \cos \psi \cos \varphi } & {\cos \theta \sin \varphi } \\ {\cos \psi \sin \theta \cos \varphi + \sin \psi \sin \phi } & {\sin \psi \sin \theta \cos \varphi - \cos \psi \sin \varphi } & {\cos \theta \cos \varphi } \\ \end{array} } \right] $$

Now consider the following definitions:

6 $$ \Delta \psi = \hat{\psi } - \psi ,\,\,\,\,\,\Delta \theta = \hat{\theta } - \theta ,\,\,\,\,\,\Delta \varphi = \hat{\varphi } - \varphi $$

where $\Delta$ defines the difference between the true and estimated value of a parameter. Utilizing Eq. (6), Eq. (4) can be written as follows:

7 $$ \begin{aligned} [{\upvarepsilon }{\mathbf{R}}^{{{\hat{\text{N}}\text{N}}}} ]^{{\text{N}}} & = [{\mathbf{E}}]^{{\text{N}}} - [{\overline{\text{T}}}]^{{{{\text{B}\hat{\text{N}}}}}} [{\text{T}}]^{{{\text{BN}}}} = [{\mathbf{E}}]^{{\text{N}}} - f^{ - 1} (\hat{\psi },\hat{\theta },\hat{\varphi }) \times f(\overset{\lower0.5em\hbox{$\smash{\scriptscriptstyle\frown}$}}{\psi } ,\overset{\lower0.5em\hbox{$\smash{\scriptscriptstyle\frown}$}}{\theta } ,\overset{\lower0.5em\hbox{$\smash{\scriptscriptstyle\frown}$}}{\varphi } ) \\ & = [{\mathbf{E}}]^{{\text{N}}} - f^{ - 1} (\psi + \Delta \psi ,\,\theta + \Delta \theta ,\varphi + \Delta \varphi ) \times f(\psi ,\theta ,\varphi ) \\ & = [{\mathbf{E}}]^{{\text{N}}} - f^{ - 1} (\hat{\psi },\hat{\theta },\hat{\varphi }) \times f(\hat{\psi } - \Delta \psi ,\hat{\theta } - \Delta \theta ,\hat{\varphi } - \Delta \varphi ) \\ \end{aligned} $$

Expanding the above equation, $[{\upvarepsilon }{\mathbf{R}}^{{{\hat{\text{N}}\text{N}}}} ]^{{\text{N}}}$ can be found as:

8 $$ \begin{aligned} & [{\upvarepsilon }{\mathbf{R}}^{{{\hat{\text{N}}\text{N}}}} ]^{{\text{N}}} = \left[ {\begin{array}{*{20}c} 0 & { - {\upvarepsilon }\psi } & {{\upvarepsilon }\theta } \\ {{\upvarepsilon }\psi } & 0 & { - {\upvarepsilon }\phi } \\ { - {\upvarepsilon }\theta } & {{\upvarepsilon }\phi } & 0 \\ \end{array} } \right] = \left[ {\begin{array}{*{20}c} 0 & { - \Delta_{3} } & {\Delta_{2} } \\ {\Delta_{1} } & 0 & { - \Delta_{1} } \\ { - \Delta_{2} } & {\Delta_{1} } & 0 \\ \end{array} } \right] \\ & \Delta_{1} = \Delta \theta \,{\text{sin}}\hat{\psi } - \Delta \phi \,{\text{cos}}\hat{\psi }\,{\text{cos}}\hat{\theta } - \Delta \psi \,\Delta \theta \,{\text{cos}}\hat{\psi } - \Delta \phi \,\Delta \psi \,{\text{cos}}\hat{\theta }\,{\text{sin}}\hat{\psi } \\ & \Delta_{2} = - \Delta \theta \,{\text{cos}}\hat{\psi } - \Delta \phi \,{\text{cos}}\hat{\theta }\,{\text{sin}}\hat{\psi } - \Delta \phi \,\Delta \theta \,{\text{sin}}\hat{\psi }\,{\text{sin}}\hat{\theta } \\ & \Delta_{3} = \Delta \phi \,{\text{sin}}\hat{\theta } - \Delta \psi - \Delta \phi \,\Delta \theta \,{\text{cos}}^{{2}} \hat{\psi }\,{\text{cos}}\hat{\theta } - \Delta \phi \,\Delta \psi \,\Delta \theta \,{\text{cos}}\hat{\psi }\,{\text{cos}}\hat{\theta }\,{\text{sin}}\hat{\psi } \\ \end{aligned} $$

The above equation can be written as Eqs. (9) or (10):

9 $$ \begin{aligned} & {\upvarepsilon }\phi = \Delta \theta \,{\text{sin}}\hat{\psi } - \Delta \phi \,{\text{cos}}\hat{\psi }\,{\text{cos}}\hat{\theta } - \Delta \psi \,\Delta \theta \,{\text{cos}}\hat{\psi } - \Delta \phi \,\Delta \psi \,{\text{cos}}\hat{\theta }\,{\text{sin}}\hat{\psi } \\ & {\upvarepsilon }\theta = - \Delta \theta \,{\text{cos}}\hat{\psi } - \Delta \phi \,{\text{cos}}\hat{\theta }\,{\text{sin}}\hat{\psi } - \Delta \phi \,\Delta \theta \,{\text{sin}}\hat{\psi }\,{\text{sin}}\hat{\theta } \\ & {\upvarepsilon }\psi = \Delta \phi \,{\text{sin}}\hat{\theta } - \Delta \psi - \Delta \phi \,\Delta \theta \,{\text{cos}}^{{2}} \hat{\psi }\,{\text{cos}}\hat{\theta } - \Delta \phi \,\Delta \psi \,\Delta \theta \,{\text{cos}}\hat{\psi }\,{\text{cos}}\hat{\theta }\,{\text{sin}}\hat{\psi } \\ \end{aligned} $$

10 $$ \begin{aligned} & {\upvarepsilon }\phi = \Delta \theta \,{\text{sin}}\overset{\lower0.5em\hbox{$\smash{\scriptscriptstyle\frown}$}}{\psi } - \Delta \phi \,{\text{cos}}\overset{\lower0.5em\hbox{$\smash{\scriptscriptstyle\frown}$}}{\psi } \,{\text{cos}}\overset{\lower0.5em\hbox{$\smash{\scriptscriptstyle\frown}$}}{\theta } - \Delta \psi \,\Delta \theta \,{\text{cos}}\overset{\lower0.5em\hbox{$\smash{\scriptscriptstyle\frown}$}}{\psi } - \Delta \phi \,\Delta \psi \,{\text{cos}}\overset{\lower0.5em\hbox{$\smash{\scriptscriptstyle\frown}$}}{\theta } \,{\text{sin}}\overset{\lower0.5em\hbox{$\smash{\scriptscriptstyle\frown}$}}{\psi } \\ & {\upvarepsilon }\theta = - \Delta \theta \,{\text{cos}}\overset{\lower0.5em\hbox{$\smash{\scriptscriptstyle\frown}$}}{\psi } - \Delta \phi \,{\text{cos}}\overset{\lower0.5em\hbox{$\smash{\scriptscriptstyle\frown}$}}{\theta } \,{\text{sin}}\overset{\lower0.5em\hbox{$\smash{\scriptscriptstyle\frown}$}}{\psi } - \Delta \phi \,\Delta \theta \,{\text{sin}}\overset{\lower0.5em\hbox{$\smash{\scriptscriptstyle\frown}$}}{\psi } \,{\text{sin}}\overset{\lower0.5em\hbox{$\smash{\scriptscriptstyle\frown}$}}{\theta } \\ & {\upvarepsilon }\psi = \Delta \phi \,{\text{sin}}\overset{\lower0.5em\hbox{$\smash{\scriptscriptstyle\frown}$}}{\theta } - \Delta \psi - \Delta \phi \,\Delta \theta \,{\text{cos}}^{{2}} \overset{\lower0.5em\hbox{$\smash{\scriptscriptstyle\frown}$}}{\psi } \,{\text{cos}}\overset{\lower0.5em\hbox{$\smash{\scriptscriptstyle\frown}$}}{\theta } - \Delta \phi \,\Delta \psi \,\Delta \theta \,{\text{cos}}\overset{\lower0.5em\hbox{$\smash{\scriptscriptstyle\frown}$}}{\psi } \,{\text{cos}}\overset{\lower0.5em\hbox{$\smash{\scriptscriptstyle\frown}$}}{\theta } \,{\text{sin}}\overset{\lower0.5em\hbox{$\smash{\scriptscriptstyle\frown}$}}{\psi } \\ \end{aligned} $$

According to Eq. (6), Eq. (10) is rewritten as follows:

11 $$ \begin{aligned} & {\upvarepsilon }\phi = (\hat{\theta } - \overset{\lower0.5em\hbox{$\smash{\scriptscriptstyle\frown}$}}{\theta } )\,{\text{sin}}\overset{\lower0.5em\hbox{$\smash{\scriptscriptstyle\frown}$}}{\psi } - (\hat{\phi } - \overset{\lower0.5em\hbox{$\smash{\scriptscriptstyle\frown}$}}{\phi } )\,{\text{cos}}\overset{\lower0.5em\hbox{$\smash{\scriptscriptstyle\frown}$}}{\psi } \,{\text{cos}}\overset{\lower0.5em\hbox{$\smash{\scriptscriptstyle\frown}$}}{\theta } - (\hat{\psi } - \overset{\lower0.5em\hbox{$\smash{\scriptscriptstyle\frown}$}}{\psi } )\,(\hat{\theta } - \overset{\lower0.5em\hbox{$\smash{\scriptscriptstyle\frown}$}}{\theta } )\,{\text{cos}}\overset{\lower0.5em\hbox{$\smash{\scriptscriptstyle\frown}$}}{\psi } - (\hat{\phi } - \overset{\lower0.5em\hbox{$\smash{\scriptscriptstyle\frown}$}}{\phi } )\,(\hat{\psi } - \overset{\lower0.5em\hbox{$\smash{\scriptscriptstyle\frown}$}}{\psi } )\,{\text{cos}}\overset{\lower0.5em\hbox{$\smash{\scriptscriptstyle\frown}$}}{\theta } \,{\text{sin}}\overset{\lower0.5em\hbox{$\smash{\scriptscriptstyle\frown}$}}{\psi } \\ & {\upvarepsilon }\theta = - (\hat{\theta } - \overset{\lower0.5em\hbox{$\smash{\scriptscriptstyle\frown}$}}{\theta } )\,{\text{cos}}\overset{\lower0.5em\hbox{$\smash{\scriptscriptstyle\frown}$}}{\psi } - (\hat{\phi } - \overset{\lower0.5em\hbox{$\smash{\scriptscriptstyle\frown}$}}{\phi } )\,{\text{cos}}\overset{\lower0.5em\hbox{$\smash{\scriptscriptstyle\frown}$}}{\theta } \,{\text{sin}}\overset{\lower0.5em\hbox{$\smash{\scriptscriptstyle\frown}$}}{\psi } - (\hat{\phi } - \overset{\lower0.5em\hbox{$\smash{\scriptscriptstyle\frown}$}}{\phi } )\,(\hat{\theta } - \overset{\lower0.5em\hbox{$\smash{\scriptscriptstyle\frown}$}}{\theta } )\,{\text{sin}}\overset{\lower0.5em\hbox{$\smash{\scriptscriptstyle\frown}$}}{\psi } \,{\text{sin}}\overset{\lower0.5em\hbox{$\smash{\scriptscriptstyle\frown}$}}{\theta } \\ & {\upvarepsilon }\psi = (\hat{\phi } - \overset{\lower0.5em\hbox{$\smash{\scriptscriptstyle\frown}$}}{\phi } )\,{\text{sin}}\overset{\lower0.5em\hbox{$\smash{\scriptscriptstyle\frown}$}}{\theta } - (\hat{\psi } - \overset{\lower0.5em\hbox{$\smash{\scriptscriptstyle\frown}$}}{\psi } ) - (\hat{\phi } - \overset{\lower0.5em\hbox{$\smash{\scriptscriptstyle\frown}$}}{\phi } )\,(\hat{\theta } - \overset{\lower0.5em\hbox{$\smash{\scriptscriptstyle\frown}$}}{\theta } )\,{\text{cos}}^{{2}} \overset{\lower0.5em\hbox{$\smash{\scriptscriptstyle\frown}$}}{\psi } \,{\text{cos}}\overset{\lower0.5em\hbox{$\smash{\scriptscriptstyle\frown}$}}{\theta } - (\hat{\phi } - \overset{\lower0.5em\hbox{$\smash{\scriptscriptstyle\frown}$}}{\phi } )\,(\hat{\psi } - \overset{\lower0.5em\hbox{$\smash{\scriptscriptstyle\frown}$}}{\psi } )\,(\hat{\theta } - \overset{\lower0.5em\hbox{$\smash{\scriptscriptstyle\frown}$}}{\theta } )\,{\text{cos}}\overset{\lower0.5em\hbox{$\smash{\scriptscriptstyle\frown}$}}{\psi } \,{\text{cos}}\overset{\lower0.5em\hbox{$\smash{\scriptscriptstyle\frown}$}}{\theta } \,{\text{sin}}\overset{\lower0.5em\hbox{$\smash{\scriptscriptstyle\frown}$}}{\psi } \\ \end{aligned} $$

The above equation demonstrates the relationship between the small rotation angles and the Euler angles. The expanded form of Eq. (11) is also as follows:

12 $$ \begin{aligned}   \varepsilon \phi  &  = \hat{\theta }\,\sin \overset{\lower0.5em\hbox{$\smash{\scriptscriptstyle\frown}$}}{\psi }  - \overset{\lower0.5em\hbox{$\smash{\scriptscriptstyle\frown}$}}{\theta } \,\sin \overset{\lower0.5em\hbox{$\smash{\scriptscriptstyle\frown}$}}{\psi }  - \hat{\phi }\,\cos \overset{\lower0.5em\hbox{$\smash{\scriptscriptstyle\frown}$}}{\psi } \,\cos \overset{\lower0.5em\hbox{$\smash{\scriptscriptstyle\frown}$}}{\theta }  + \overset{\lower0.5em\hbox{$\smash{\scriptscriptstyle\frown}$}}{\phi } \,\cos \overset{\lower0.5em\hbox{$\smash{\scriptscriptstyle\frown}$}}{\psi } \,\cos \overset{\lower0.5em\hbox{$\smash{\scriptscriptstyle\frown}$}}{\theta }  + \hat{\psi }\overset{\lower0.5em\hbox{$\smash{\scriptscriptstyle\frown}$}}{\theta } \,\cos \overset{\lower0.5em\hbox{$\smash{\scriptscriptstyle\frown}$}}{\psi }  - \hat{\psi }\hat{\theta }\,\cos \overset{\lower0.5em\hbox{$\smash{\scriptscriptstyle\frown}$}}{\psi }  \\     & \quad  + \overset{\lower0.5em\hbox{$\smash{\scriptscriptstyle\frown}$}}{\psi } \hat{\theta }\,\cos \overset{\lower0.5em\hbox{$\smash{\scriptscriptstyle\frown}$}}{\psi }  - \overset{\lower0.5em\hbox{$\smash{\scriptscriptstyle\frown}$}}{\psi } \overset{\lower0.5em\hbox{$\smash{\scriptscriptstyle\frown}$}}{\theta } \,\cos \overset{\lower0.5em\hbox{$\smash{\scriptscriptstyle\frown}$}}{\psi }  + \hat{\phi }\overset{\lower0.5em\hbox{$\smash{\scriptscriptstyle\frown}$}}{\psi } \,\cos \overset{\lower0.5em\hbox{$\smash{\scriptscriptstyle\frown}$}}{\theta } \,\sin \overset{\lower0.5em\hbox{$\smash{\scriptscriptstyle\frown}$}}{\psi }  - \hat{\phi }\hat{\psi }\,\cos \overset{\lower0.5em\hbox{$\smash{\scriptscriptstyle\frown}$}}{\theta } \,\sin \overset{\lower0.5em\hbox{$\smash{\scriptscriptstyle\frown}$}}{\psi }  + \overset{\lower0.5em\hbox{$\smash{\scriptscriptstyle\frown}$}}{\phi } \hat{\psi }\,\cos \overset{\lower0.5em\hbox{$\smash{\scriptscriptstyle\frown}$}}{\theta } \,\sin \overset{\lower0.5em\hbox{$\smash{\scriptscriptstyle\frown}$}}{\psi }  - \overset{\lower0.5em\hbox{$\smash{\scriptscriptstyle\frown}$}}{\phi } \overset{\lower0.5em\hbox{$\smash{\scriptscriptstyle\frown}$}}{\psi } \,\cos \overset{\lower0.5em\hbox{$\smash{\scriptscriptstyle\frown}$}}{\theta } \,\sin \overset{\lower0.5em\hbox{$\smash{\scriptscriptstyle\frown}$}}{\psi }  \\    \varepsilon \theta  &  =  - \hat{\theta }\,\cos \overset{\lower0.5em\hbox{$\smash{\scriptscriptstyle\frown}$}}{\psi }  + \overset{\lower0.5em\hbox{$\smash{\scriptscriptstyle\frown}$}}{\theta } \,\cos \overset{\lower0.5em\hbox{$\smash{\scriptscriptstyle\frown}$}}{\psi }  - \hat{\phi }\,\cos \overset{\lower0.5em\hbox{$\smash{\scriptscriptstyle\frown}$}}{\theta } \,\sin \overset{\lower0.5em\hbox{$\smash{\scriptscriptstyle\frown}$}}{\psi }  + \overset{\lower0.5em\hbox{$\smash{\scriptscriptstyle\frown}$}}{\phi } \,\cos \overset{\lower0.5em\hbox{$\smash{\scriptscriptstyle\frown}$}}{\theta } \,\sin \overset{\lower0.5em\hbox{$\smash{\scriptscriptstyle\frown}$}}{\psi }  + \hat{\phi }\overset{\lower0.5em\hbox{$\smash{\scriptscriptstyle\frown}$}}{\theta } \,\sin \overset{\lower0.5em\hbox{$\smash{\scriptscriptstyle\frown}$}}{\psi } \,\sin \overset{\lower0.5em\hbox{$\smash{\scriptscriptstyle\frown}$}}{\theta }  \\     & \quad  - \hat{\phi }\hat{\theta }\,\sin \overset{\lower0.5em\hbox{$\smash{\scriptscriptstyle\frown}$}}{\psi } \,\sin \overset{\lower0.5em\hbox{$\smash{\scriptscriptstyle\frown}$}}{\theta }  + \overset{\lower0.5em\hbox{$\smash{\scriptscriptstyle\frown}$}}{\phi } \hat{\theta }\,\sin \overset{\lower0.5em\hbox{$\smash{\scriptscriptstyle\frown}$}}{\psi } \,\sin \overset{\lower0.5em\hbox{$\smash{\scriptscriptstyle\frown}$}}{\theta }  - \overset{\lower0.5em\hbox{$\smash{\scriptscriptstyle\frown}$}}{\phi } \overset{\lower0.5em\hbox{$\smash{\scriptscriptstyle\frown}$}}{\theta } \,\sin \overset{\lower0.5em\hbox{$\smash{\scriptscriptstyle\frown}$}}{\psi } \,\sin \overset{\lower0.5em\hbox{$\smash{\scriptscriptstyle\frown}$}}{\theta }  \\    \varepsilon \psi  &  = \hat{\phi }\,\sin \overset{\lower0.5em\hbox{$\smash{\scriptscriptstyle\frown}$}}{\theta }  - \overset{\lower0.5em\hbox{$\smash{\scriptscriptstyle\frown}$}}{\phi } \,\sin \overset{\lower0.5em\hbox{$\smash{\scriptscriptstyle\frown}$}}{\theta }  - \hat{\psi } + \overset{\lower0.5em\hbox{$\smash{\scriptscriptstyle\frown}$}}{\psi }  + \hat{\phi }\overset{\lower0.5em\hbox{$\smash{\scriptscriptstyle\frown}$}}{\theta } \,\cos ^{2} \overset{\lower0.5em\hbox{$\smash{\scriptscriptstyle\frown}$}}{\psi } \,\cos \overset{\lower0.5em\hbox{$\smash{\scriptscriptstyle\frown}$}}{\theta }  - \hat{\phi }\hat{\theta }\,\cos ^{2} \overset{\lower0.5em\hbox{$\smash{\scriptscriptstyle\frown}$}}{\psi } \,\cos \overset{\lower0.5em\hbox{$\smash{\scriptscriptstyle\frown}$}}{\theta }  + \overset{\lower0.5em\hbox{$\smash{\scriptscriptstyle\frown}$}}{\phi } \hat{\theta }\,\cos ^{2} \overset{\lower0.5em\hbox{$\smash{\scriptscriptstyle\frown}$}}{\psi } \,\cos \overset{\lower0.5em\hbox{$\smash{\scriptscriptstyle\frown}$}}{\theta }  - \overset{\lower0.5em\hbox{$\smash{\scriptscriptstyle\frown}$}}{\phi } \overset{\lower0.5em\hbox{$\smash{\scriptscriptstyle\frown}$}}{\theta } \,\cos ^{2} \overset{\lower0.5em\hbox{$\smash{\scriptscriptstyle\frown}$}}{\psi } \,\cos \overset{\lower0.5em\hbox{$\smash{\scriptscriptstyle\frown}$}}{\theta }  \\     & \quad  + \hat{\phi }\hat{\psi }\overset{\lower0.5em\hbox{$\smash{\scriptscriptstyle\frown}$}}{\theta } \,\cos \overset{\lower0.5em\hbox{$\smash{\scriptscriptstyle\frown}$}}{\psi } \,\cos \overset{\lower0.5em\hbox{$\smash{\scriptscriptstyle\frown}$}}{\theta } \,\sin \overset{\lower0.5em\hbox{$\smash{\scriptscriptstyle\frown}$}}{\psi }  - \hat{\phi }\hat{\psi }\hat{\theta }\,\cos \overset{\lower0.5em\hbox{$\smash{\scriptscriptstyle\frown}$}}{\psi } \,\cos \overset{\lower0.5em\hbox{$\smash{\scriptscriptstyle\frown}$}}{\theta } \,\sin \overset{\lower0.5em\hbox{$\smash{\scriptscriptstyle\frown}$}}{\psi }  + \hat{\phi }\overset{\lower0.5em\hbox{$\smash{\scriptscriptstyle\frown}$}}{\psi } \hat{\theta }\,\cos \overset{\lower0.5em\hbox{$\smash{\scriptscriptstyle\frown}$}}{\psi } \,\cos \overset{\lower0.5em\hbox{$\smash{\scriptscriptstyle\frown}$}}{\theta } \,\sin \overset{\lower0.5em\hbox{$\smash{\scriptscriptstyle\frown}$}}{\psi }  + \overset{\lower0.5em\hbox{$\smash{\scriptscriptstyle\frown}$}}{\phi } \hat{\psi }\hat{\theta }\,\cos \overset{\lower0.5em\hbox{$\smash{\scriptscriptstyle\frown}$}}{\psi } \,\cos \overset{\lower0.5em\hbox{$\smash{\scriptscriptstyle\frown}$}}{\theta } \,\sin \overset{\lower0.5em\hbox{$\smash{\scriptscriptstyle\frown}$}}{\psi }  \\     & \quad  - \hat{\phi }\overset{\lower0.5em\hbox{$\smash{\scriptscriptstyle\frown}$}}{\psi } \overset{\lower0.5em\hbox{$\smash{\scriptscriptstyle\frown}$}}{\theta } \,\cos \overset{\lower0.5em\hbox{$\smash{\scriptscriptstyle\frown}$}}{\psi } \,\cos \overset{\lower0.5em\hbox{$\smash{\scriptscriptstyle\frown}$}}{\theta } \,\sin \overset{\lower0.5em\hbox{$\smash{\scriptscriptstyle\frown}$}}{\psi }  - \overset{\lower0.5em\hbox{$\smash{\scriptscriptstyle\frown}$}}{\phi } \hat{\psi }\overset{\lower0.5em\hbox{$\smash{\scriptscriptstyle\frown}$}}{\theta } \,\cos \overset{\lower0.5em\hbox{$\smash{\scriptscriptstyle\frown}$}}{\psi } \,\cos \overset{\lower0.5em\hbox{$\smash{\scriptscriptstyle\frown}$}}{\theta } \,\sin \overset{\lower0.5em\hbox{$\smash{\scriptscriptstyle\frown}$}}{\psi }  - \overset{\lower0.5em\hbox{$\smash{\scriptscriptstyle\frown}$}}{\phi } \overset{\lower0.5em\hbox{$\smash{\scriptscriptstyle\frown}$}}{\psi } \hat{\theta }\,\cos \overset{\lower0.5em\hbox{$\smash{\scriptscriptstyle\frown}$}}{\psi } \,\cos \overset{\lower0.5em\hbox{$\smash{\scriptscriptstyle\frown}$}}{\theta } \,\sin \overset{\lower0.5em\hbox{$\smash{\scriptscriptstyle\frown}$}}{\psi }  + \overset{\lower0.5em\hbox{$\smash{\scriptscriptstyle\frown}$}}{\phi } \overset{\lower0.5em\hbox{$\smash{\scriptscriptstyle\frown}$}}{\psi } \overset{\lower0.5em\hbox{$\smash{\scriptscriptstyle\frown}$}}{\theta } \,\cos \overset{\lower0.5em\hbox{$\smash{\scriptscriptstyle\frown}$}}{\psi } \,\cos \overset{\lower0.5em\hbox{$\smash{\scriptscriptstyle\frown}$}}{\theta } \,\sin \overset{\lower0.5em\hbox{$\smash{\scriptscriptstyle\frown}$}}{\psi }  \\  \end{aligned}    $$

In the next section, the above equation is used to extract the error of IMU sensors in a stand-alone mode.

## The proposed algorithm formulation

Consider the set of velocity and attitude navigation error equations. These equations are given in Ref.^25^ as follows:

13 $$ \begin{aligned} \varepsilon \dot{v}_{{\text{n}}} & = {\text{T}}_{{{11}}}^{{{\text{NB}}}} \delta f_{{\text{x}}} + {\text{T}}_{{{12}}}^{{{\text{NB}}}} \delta f_{{\text{y}}} + {\text{T}}_{{{13}}}^{{{\text{NB}}}} \delta f_{{\text{z}}} + f_{{\text{e}}} \varepsilon \psi - f_{{\text{d}}} \varepsilon \theta - \left( {\frac{{v_{{\text{e}}}^{2} \sec^{2} \lambda }}{{{\text{R}}_{{\text{e}}} }} + 2\omega_{{\text{n}}} v_{{\text{e}}} } \right)\varepsilon \lambda \\ & \quad + \frac{{(v_{{\text{e}}}^{2} \tan \lambda - v_{{\text{n}}} v_{{\text{d}}} )}}{{{\text{R}}_{{\text{e}}}^{2} }}\varepsilon h + \frac{{v_{{\text{d}}} }}{{{\text{R}}_{{\text{e}}} }}\varepsilon v_{{\text{n}}} + \left( {2\omega_{{\text{d}}} - \frac{{2v_{{\text{e}}} \tan \lambda }}{{{\text{R}}_{{\text{e}}} }}} \right)\varepsilon v_{{\text{e}}} + \frac{{v_{{\text{n}}} }}{{{\text{R}}_{{\text{e}}} }}\varepsilon v_{{\text{d}}} \\ \varepsilon \dot{v}_{{\text{e}}} & = {\text{T}}_{{{21}}}^{{{\text{NB}}}} \delta f_{{\text{x}}} + {\text{T}}_{{{22}}}^{{{\text{NB}}}} \delta f_{{\text{y}}} + {\text{T}}_{{{23}}}^{{{\text{NB}}}} \delta f_{{\text{z}}} - f_{{\text{n}}} \varepsilon \psi + f_{{\text{d}}} \varepsilon \phi + \left( {\frac{{v_{{\text{e}}} v_{{\text{n}}} \sec^{2} \lambda }}{{{\text{R}}_{{\text{e}}} }} + 2\omega_{{\text{n}}} v_{{\text{n}}} + 2\omega_{{\text{d}}} v_{{\text{d}}} } \right)\varepsilon \lambda \\ & \quad - \frac{{(v_{{\text{e}}} v_{{\text{n}}} \tan \lambda + v_{{\text{e}}} v_{{\text{d}}} )}}{{{\text{R}}_{{\text{e}}}^{2} }}\varepsilon h + \left( {\frac{{v_{{\text{e}}} }}{{{\text{R}}_{{\text{e}}} }} + 2\omega_{{\text{n}}} } \right)\varepsilon v_{{\text{d}}} + \left( {\frac{{v_{{\text{e}}} \tan \lambda }}{{{\text{R}}_{{\text{e}}} }} - 2\omega_{{\text{d}}} } \right)\varepsilon v_{{\text{n}}} + \frac{{(v_{{\text{n}}} \tan \lambda + v_{{\text{d}}} )}}{{{\text{R}}_{{\text{e}}} }}\varepsilon v_{{\text{e}}} \\ \varepsilon \dot{v}_{{\text{d}}} & = {\text{T}}_{{{31}}}^{{{\text{NB}}}} \delta f_{{\text{x}}} + {\text{T}}_{{{32}}}^{{{\text{NB}}}} \delta f_{{\text{y}}} + {\text{T}}_{{{33}}}^{{{\text{NB}}}} \delta f_{{\text{z}}} + f_{{\text{n}}} \varepsilon \theta - f_{{\text{e}}} \varepsilon \phi - 2\omega_{{\text{d}}} v_{{\text{e}}} \varepsilon \lambda \\ & \quad + \frac{{(v_{{\text{e}}}^{2} + v_{{\text{n}}}^{2} )}}{{{\text{R}}_{{\text{e}}}^{2} }}\varepsilon h + \delta {\text{g}} - \frac{{2v_{{\text{n}}} }}{{{\text{R}}_{{\text{e}}} }}\varepsilon v_{{\text{n}}} - \left( {\frac{{2v_{{\text{e}}} }}{{{\text{R}}_{{\text{e}}} }} + 2\omega_{{\text{n}}} } \right)\varepsilon v_{{\text{e}}} \\ \end{aligned} $$

14 $$ \begin{aligned} \varepsilon \dot{\phi } & = \omega_{{\text{d}}} \varepsilon \lambda - \frac{{v_{{\text{e}}} }}{{{\text{R}}_{{\text{e}}}^{2} }}\varepsilon h + \frac{1}{{{\text{R}}_{{\text{e}}} }}\varepsilon v_{{\text{e}}} + \left( {\omega_{{\text{d}}} - \frac{{v_{{\text{e}}} \tan \lambda }}{{{\text{R}}_{{\text{e}}} }}} \right)\varepsilon \theta + \frac{{v_{{\text{n}}} }}{{{\text{R}}_{{\text{e}}} }}\varepsilon \psi \\ & \quad - {\text{T}}_{{{11}}}^{{{\text{NB}}}} \delta \omega_{{\text{x}}} - {\text{T}}_{{{12}}}^{{{\text{NB}}}} \delta \omega_{{\text{y}}} - {\text{T}}_{{{13}}}^{{{\text{NB}}}} \delta \omega_{{\text{z}}} \\ \varepsilon \dot{\theta } & = \frac{{v_{{\text{n}}} }}{{{\text{R}}_{{\text{e}}}^{2} }}\varepsilon h - \frac{1}{{{\text{R}}_{{\text{e}}} }}\varepsilon v_{{\text{n}}} + \left( {\frac{{v_{{\text{e}}} \tan \lambda }}{{{\text{R}}_{{\text{e}}} }} - \omega_{{\text{d}}} } \right)\varepsilon \phi + \left( {\omega_{{\text{n}}} + \frac{{v_{{\text{e}}} }}{{{\text{R}}_{{\text{e}}} }}} \right)\varepsilon \psi \\ & \quad - {\text{T}}_{{{21}}}^{{{\text{NB}}}} {\updelta }\omega_{{\text{x}}} - {\text{T}}_{{{22}}}^{{{\text{NB}}}} {\updelta }\omega_{{\text{y}}} - {\text{T}}_{{{23}}}^{{{\text{NB}}}} {\updelta }\omega_{{\text{z}}} \\ \varepsilon \dot{\psi } & = - \left( {\omega_{{\text{n}}} + \frac{{v_{{\text{e}}} \sec^{2} \lambda }}{{{\text{R}}_{{\text{e}}} }}} \right)\varepsilon \lambda + \frac{{v_{{\text{e}}} \tan \lambda }}{{{\text{R}}_{{\text{e}}}^{2} }}\varepsilon h - \frac{\tan \lambda }{{{\text{R}}_{{\text{e}}} }}\varepsilon v_{{\text{e}}} - \frac{{v_{{\text{n}}} }}{{{\text{R}}_{{\text{e}}} }}\varepsilon \phi - \left( {\omega_{{\text{n}}} + \frac{{v_{{\text{e}}} }}{{{\text{R}}_{{\text{e}}} }}} \right)\varepsilon \theta \\ & \quad - {\text{T}}_{{{31}}}^{{{\text{NB}}}} \delta \omega_{{\text{x}}} - {\text{T}}_{{{32}}}^{{{\text{NB}}}} \delta \omega_{{\text{y}}} - {\text{T}}_{{{33}}}^{{{\text{NB}}}} \delta \omega_{{\text{z}}} \\ \end{aligned} $$

where $\lambda$, $\ell$, $v_{{\text{n}}}$, $v_{{\text{e}}}$ and $v_{{\text{d}}}$ denote latitude, longitude, and north, east, and down terrestrial velocities, respectively. ${\text{R}}_{{\text{e}}}$ is the radius of the Earth and ${\upvarepsilon }$ is the perturbation operator. The attitude error between the true and computed navigation frames is defined by ${\upvarepsilon }\phi$, ${\upvarepsilon }\theta$ and ${\upvarepsilon }\psi$. Also, ${\upomega }^{{{\text{EI}}}}$ is the Earth’s angular velocity and ${\upomega }_{{\text{n}}}$, ${\upomega }_{{\text{d}}}$, $f_{{\text{n}}}$, $f_{{\text{e}}}$ and $f_{{\text{d}}}$ are defined as follows:

15 $$ [{{\varvec{\upomega}}}^{{{\text{EI}}}} ]^{{\text{N}}} = \left[ {\begin{array}{*{20}c} {{\upomega }^{{{\text{EI}}}} \cos \lambda } \\ {0} \\ { - {\upomega }^{{{\text{EI}}}} \sin \lambda } \\ \end{array} } \right] = \left[ {\begin{array}{*{20}c} {{\upomega }_{{\text{n}}} } \\ {0} \\ {{\upomega }_{{\text{d}}} } \\ \end{array} } \right] $$

16 $$ {[}{\mathbf{a}}_{{\text{B}}}^{{\text{I}}} {]}^{{\text{N}}} = \left[ {\begin{array}{*{20}c} {f_{{\text{n}}} } \\ {f_{{\text{e}}} } \\ {f_{{\text{d}}} } \\ \end{array} } \right] = [{\text{T}}]^{{{\text{NB}}}} \left[ {\begin{array}{*{20}c} {f_{{\text{x}}} } \\ {f_{{\text{y}}} } \\ {f_{{\text{z}}} } \\ \end{array} } \right] $$

Applying stationary conditions ($$[{\mathbf{v}}_{{\text{B}}}^{{\text{E}}} ]^{{\text{N}}} = [\begin{array}{*{20}c} 0 & 0 & 0 \\ \end{array} ]^{{\text{T}}}$$ and $$[{\mathbf{f}}]^{{\text{N}}} = [ - {\mathbf{g}}]^{{\text{N}}} = [\begin{array}{*{20}c} 0 & 0 & { - {\text{g}}} \\ \end{array} ]^{{\text{T}}}$$) to Eqs. (13)–(14), assuming spherical Earth, neglecting position parameters (${\upvarepsilon }\lambda$ and ${\upvarepsilon }h$), ignoring the gravity model error (${\delta g} = {0}$), navigation error equations are simplified as follows:

17 $$ \begin{aligned} & {\upvarepsilon }\dot{v}_{{\text{n}}} = {\updelta }f_{{\text{n}}} + 2{\upomega }_{{\text{d}}} {\upvarepsilon }v_{{\text{e}}} + {{\text{g}\varepsilon }}\theta \\ & {\upvarepsilon }\dot{v}_{{\text{e}}} = {\updelta }f_{{\text{e}}} - 2{\upomega }_{{\text{d}}} {\upvarepsilon }v_{{\text{n}}} + 2{\upomega }_{{\text{n}}} {\upvarepsilon }v_{{\text{d}}} - {{\text{g}\varepsilon }}\phi \\ & {\upvarepsilon }\dot{v}_{{\text{d}}} = {\updelta }f_{{\text{d}}} - 2{\upomega }_{{\text{n}}} {\upvarepsilon }v_{{\text{e}}} \\ & {\upvarepsilon }\dot{\phi } = - {\updelta }\omega_{{\text{n}}} + \left( {{1 \mathord{\left/ {\vphantom {1 {{\text{R}}_{{\text{e}}} }}} \right. \kern-0pt} {{\text{R}}_{{\text{e}}} }}} \right){\upvarepsilon }v_{{\text{e}}} + {\upomega }_{{\text{d}}} {\upvarepsilon }\theta \\ & {\upvarepsilon }\dot{\theta } = - {\updelta }\omega_{{\text{e}}} - \left( {{1 \mathord{\left/ {\vphantom {1 {{\text{R}}_{{\text{e}}} }}} \right. \kern-0pt} {{\text{R}}_{{\text{e}}} }}} \right){\upvarepsilon }v_{{\text{n}}} - {\upomega }_{{\text{d}}} {\upvarepsilon }\phi + \,{\upomega }_{{\text{n}}} {\upvarepsilon }\psi \\ & {\upvarepsilon }\dot{\psi } = - {\updelta }\omega_{{\text{d}}} - \left( {{1 \mathord{\left/ {\vphantom {1 {{\text{R}}_{{\text{e}}} }}} \right. \kern-0pt} {{\text{R}}_{{\text{e}}} }}} \right)\tan \lambda {\upvarepsilon }v_{{\text{e}}} - {\upomega }_{{\text{n}}} {\upvarepsilon }\theta \\ \end{aligned} $$

### IMU calibration

Calibration of inertial sensors is an important procedure which affects the performance of navigation products. Due to stochastic nature of sensor’s noises, factory calibration is not a perfect process. Performing in-motion or in-run calibration may increase the IMU accuracy. In this section, a new method for IMU calibration is proposed and its application in initial alignment is discussed.

The problem of IMU calibration is investigated by many researchers. Reference^26^ explores the present state and upcoming directions of MEMS inertial sensor calibration technology. It discusses the existing advancements in this field while shedding light on potential future trends. The authors analyze the significance of accurate calibration for MEMS sensors and highlight the challenges involved. Reference^27^ presents a systematic method for calibrating inertial sensors on gravity recovery satellites. The authors introduce a comprehensive approach for achieving accurate calibration, emphasizing its importance for gravity recovery missions. The study outlines the calibration process and its benefits. This work contributes to the enhancement of satellite-based gravity recovery systems through robust inertial sensor calibration techniques.

Reference^28^ introduces a novel self-calibration method for inertial measurement units (IMUs) utilizing distributed inertial sensors. The authors propose an innovative approach that leverages multiple sensors to calibrate IMUs autonomously. This method enhances the accuracy and reliability of IMU measurements by exploiting distributed information. The study outlines the self-calibration process and its benefits, highlighting its potential to improve IMU performance in various applications.

Reference^29^ focuses on the extrinsic calibration of visual and inertial sensors for autonomous vehicles. The authors present a method for accurately calibrating the relative positions and orientations of both types of sensors to facilitate robust perception and navigation. The study emphasizes the importance of precise extrinsic calibration in enhancing the perception capabilities of autonomous vehicles. Reference^30^ presents a self-calibration method for arrays of inertial sensors. The authors propose an innovative approach that enables the automatic calibration of multiple inertial sensors within an array. This method aims to enhance the accuracy and reliability of sensor measurements by leveraging inter-sensor correlations and spatial relationships.

### Accelerometers calibration

Consider the velocity error in Eq. (17) and assume that ${\upvarepsilon }\theta = {\upvarepsilon }\phi = 0$, the following simplified equations are obtained:

18 $$ \begin{aligned} & {\upvarepsilon }\dot{v}_{{\text{n}}} = {\updelta }f_{{\text{n}}} + 2{\upomega }_{{\text{d}}} {\upvarepsilon }v_{{\text{e}}} \\ & {\upvarepsilon }\dot{v}_{{\text{e}}} = {\updelta }f_{{\text{e}}} - 2{\upomega }_{{\text{d}}} {\upvarepsilon }v_{{\text{n}}} + 2{\upomega }_{{\text{n}}} {\upvarepsilon }v_{{\text{d}}} \\ & {\upvarepsilon }\dot{v}_{{\text{d}}} = {\updelta }f_{{\text{d}}} - 2{\upomega }_{{\text{n}}} {\upvarepsilon }v_{{\text{e}}} \\ \end{aligned} $$

Integrating the above equations in the time domain leads to:

19 $$ \begin{aligned} & \int_{0}^{t} {{\upvarepsilon }\dot{v}_{{\text{n}}} {\text{d}}t} = \int_{0}^{t} {{\updelta }f_{{\text{n}}} {\text{d}}t} + \int_{0}^{t} {2{\upomega }_{{\text{d}}} {\upvarepsilon }v_{{\text{e}}} {\text{d}}t} \\ & \int_{0}^{t} {{\upvarepsilon }\dot{v}_{{\text{e}}} {\text{d}}t} = \int_{0}^{t} {{\updelta }f_{{\text{e}}} {\text{d}}t} - \int_{0}^{t} {2{\upomega }_{{\text{d}}} {\upvarepsilon }v_{{\text{n}}} {\text{d}}t} + \int_{0}^{t} {2{\upomega }_{{\text{n}}} {\upvarepsilon }v_{{\text{d}}} {\text{d}}t} \\ & \int_{0}^{t} {{\upvarepsilon }\dot{v}_{{\text{d}}} {\text{d}}t} = \int_{0}^{t} {{\updelta }f_{{\text{d}}} {\text{d}}t} - \int_{0}^{t} {2{\upomega }_{{\text{n}}} {\upvarepsilon }v_{{\text{e}}} {\text{d}}t} \\ \end{aligned} $$

The above equation is expanded as follows:

20 $$ \begin{aligned} & {\upvarepsilon }v_{{\text{n}}} (t) - {\upvarepsilon }v_{{\text{n}}} (0) = {\updelta }f_{{\text{n}}} (t - 0) + 2{\upomega }_{{\text{d}}} \int_{0}^{t} {{\upvarepsilon }v_{{\text{e}}} {\text{d}}t} \\ & {\upvarepsilon }v_{{\text{e}}} (t) - {\upvarepsilon }v_{{\text{e}}} (0) = {\updelta }f_{{\text{e}}} (t - 0) - 2{\upomega }_{{\text{d}}} \int_{0}^{t} {{\upvarepsilon }v_{{\text{n}}} {\text{d}}t} + 2{\upomega }_{{\text{n}}} \int_{0}^{t} {{\upvarepsilon }v_{{\text{d}}} {\text{d}}t} \\ & {\upvarepsilon }v_{{\text{d}}} (t) - {\upvarepsilon }v_{{\text{d}}} (0) = {\updelta }f_{{\text{d}}} (t - 0) - 2{\upomega }_{{\text{n}}} \int_{0}^{t} {{\upvarepsilon }v_{{\text{e}}} {\text{d}}t} \\ \end{aligned} $$

Assuming ${\upvarepsilon }v_{{\text{n}}} (0) = {\upvarepsilon }v_{{\text{e}}} (0) = {\upvarepsilon }v_{{\text{d}}} (0) = 0$, The above equation is simplified as follows:

21 $$ \begin{aligned} & {\updelta }f_{{\text{n}}} t = {\upvarepsilon }v_{{\text{n}}} (t) - 2{\upomega }_{{\text{d}}} \int_{0}^{t} {{\upvarepsilon }v_{{\text{e}}} {\text{d}}t} \\ & {\updelta }f_{{\text{e}}} t = {\upvarepsilon }v_{{\text{e}}} (t) + 2{\upomega }_{{\text{d}}} \int_{0}^{t} {{\upvarepsilon }v_{{\text{n}}} {\text{d}}t} - 2{\upomega }_{{\text{n}}} \int_{0}^{t} {{\upvarepsilon }v_{{\text{d}}} {\text{d}}t} \\ & {\updelta }f_{{\text{d}}} t = {\upvarepsilon }v_{{\text{d}}} (t) + 2{\upomega }_{{\text{n}}} \int_{0}^{t} {{\upvarepsilon }v_{{\text{e}}} {\text{d}}t} \\ \end{aligned} $$

Based on the definition of the ${\upvarepsilon }$ operator (Appendix A) and that the true velocity is zero in stationary mode, ${\upvarepsilon }v_{i} \,\,(i = {\text{n,}}\,{\text{e,}}\,{\text{d)}}$ will be simplified as:

22 $$ \begin{aligned} & {\upvarepsilon }v_{{\text{n}}} (t) = v_{{\text{n}}} (t) - 0 = v_{{\text{n}}} (t) \\ & {\upvarepsilon }v_{{\text{e}}} (t) = v_{{\text{e}}} (t) - 0 = v_{{\text{e}}} (t) \\ & {\upvarepsilon }v_{{\text{d}}} (t) = v_{{\text{d}}} (t) - 0 = v_{{\text{d}}} (t) \\ \end{aligned} $$

Based on the above relation, Eq. (21) is rewritten as follows:

23 $$ \begin{aligned} & {\updelta }f_{{\text{n}}} = \frac{{v_{{\text{n}}} (t) - 2{\upomega }_{{\text{d}}} \int_{0}^{t} {v_{{\text{e}}} (t){\text{d}}t} }}{t} \\ & {\updelta }f_{{\text{e}}} = \frac{{v_{{\text{e}}} (t) + 2{\upomega }_{{\text{d}}} \int_{0}^{t} {v_{{\text{n}}} (t){\text{d}}t - 2{\upomega }_{{\text{n}}} \int_{0}^{t} {v_{{\text{d}}} (t){\text{d}}t} } }}{t} \\ & {\updelta }f_{{\text{d}}} = \frac{{v_{{\text{d}}} (t) + 2{\upomega }_{{\text{n}}} \int_{0}^{t} {v_{{\text{e}}} (t){\text{d}}t} }}{t} \\ \end{aligned} $$

According to the above equation, the projection of accelerometers error in the navigation coordinate is obtained; these errors can be compensated, and thus the IMU accuracy is improved.

### Gyros calibration

Consider the attitude error terms in Eq. (17) and integrating them over time leads to:

24 $$ \begin{aligned} & \int_{0}^{t} {{\upvarepsilon }\dot{\phi }{\text{d}}t} = - \int_{0}^{t} {{\updelta }\omega_{{\text{n}}} {\text{d}}t} + \int_{0}^{t} {\frac{1}{{{\text{R}}_{{\text{e}}} }}{\upvarepsilon }v_{{\text{e}}} {\text{d}}t} + \int_{0}^{t} {{\upomega }_{{\text{d}}} {\upvarepsilon }\theta \,{\text{d}}t} \\ & \int_{0}^{t} {{\upvarepsilon }\dot{\theta }{\text{d}}t} = - \int_{0}^{t} {{\updelta }\omega_{{\text{e}}} {\text{d}}t} - \int_{0}^{t} {\frac{1}{{{\text{R}}_{{\text{e}}} }}{\upvarepsilon }v_{{\text{n}}} {\text{d}}t} - \int_{0}^{t} {{\upomega }_{{\text{d}}} {\upvarepsilon }\phi {\text{d}}t} + \int_{0}^{t} {{\upomega }_{{\text{n}}} {\upvarepsilon }\psi \,{\text{d}}t} \\ & \int_{0}^{t} {{\upvarepsilon }\dot{\psi }{\text{d}}t} = - \int_{0}^{t} {{\updelta }\omega_{{\text{d}}} {\text{d}}t} - \int_{0}^{t} {\frac{\tan \lambda }{{{\text{R}}_{{\text{e}}} }}{\upvarepsilon }v_{{\text{e}}} {\text{d}}t} - \int_{0}^{t} {{\upomega }_{{\text{n}}} {\upvarepsilon }\theta \,{\text{d}}t} \\ \end{aligned} $$

Equation (24) is rewritten as follows:

25 $$ {\updelta }\omega_{{\text{n}}} t = + \frac{1}{{{\text{R}}_{{\text{e}}} }}\int_{0}^{\,t} {v_{{\text{e}}} {\text{d}}t} + {\upomega }_{{\text{d}}} \int_{0}^{\,t} {\,{{[\varepsilon }}\theta ]{\text{d}}t} - {\upvarepsilon }\phi (t) + {\upvarepsilon }\phi (0) $$

26 $$ {\updelta }\omega_{{\text{e}}} t = - \frac{1}{{{\text{R}}_{{\text{e}}} }}\int_{0}^{\,t} {v_{{\text{n}}} {\text{d}}t} - {\upomega }_{{\text{d}}} \int_{0}^{\,t} {\,{{[\varepsilon }}\phi ]{\text{d}}t} + {\upomega }_{{\text{n}}} \int_{0}^{t} {\,{{[\varepsilon }}\psi ]{\text{d}}t} - {\upvarepsilon }\theta (t) + {\upvarepsilon }\theta (0) $$

27 $$ {\updelta }\omega_{{\text{d}}} t = - \frac{\tan \lambda }{{{\text{R}}_{{\text{e}}} }}\int_{0}^{t} {v_{{\text{e}}} {\text{d}}t} - {\upomega }_{{\text{n}}} \int_{0}^{t} {{{[\upvarepsilon }}\theta ]{\text{d}}t} - {\upvarepsilon }\psi (t) + {\upvarepsilon }\psi (0) $$

Now consider the following assumptions which come from the stationary condition:

28 $$ \begin{aligned} & \overset{\lower0.5em\hbox{$\smash{\scriptscriptstyle\frown}$}}{\phi } (t) = \overset{\lower0.5em\hbox{$\smash{\scriptscriptstyle\frown}$}}{\phi } (0) = {\text{constant}} \\ & \overset{\lower0.5em\hbox{$\smash{\scriptscriptstyle\frown}$}}{\theta } (t) = \overset{\lower0.5em\hbox{$\smash{\scriptscriptstyle\frown}$}}{\theta } (0) = {\text{constant}} \\ & \overset{\lower0.5em\hbox{$\smash{\scriptscriptstyle\frown}$}}{\psi } (t) = \overset{\lower0.5em\hbox{$\smash{\scriptscriptstyle\frown}$}}{\psi } (0) = {\text{constant}} \\ \end{aligned} $$

Replacing Eqs. (12) and (28) into Eq. (25) leads to:

29 $$  \begin{aligned}   {{\delta }}\omega _{{\text{n}}} t &  =  + \frac{1}{{{\text{R}}_{{\text{e}}} }}\int_{0}^{t} {v_{{\text{e}}} {\text{(}}t{\text{)d}}t}  - {{\omega }}_{{\text{d}}} {\text{cos}}\overset{\lower0.5em\hbox{$\smash{\scriptscriptstyle\frown}$}}{\psi } (0)\int_{0}^{t} {\hat{\theta }{\text{(t)d}}t}  + {{\omega }}_{{\text{d}}} {\text{cos}}\overset{\lower0.5em\hbox{$\smash{\scriptscriptstyle\frown}$}}{\psi } (0)\overset{\lower0.5em\hbox{$\smash{\scriptscriptstyle\frown}$}}{\theta } (0)\int_{0}^{t} {{\text{d}}t}  \\     & \quad  - {{\omega }}_{{\text{d}}} {\text{cos}}\overset{\lower0.5em\hbox{$\smash{\scriptscriptstyle\frown}$}}{\theta } (0){\text{sin}}\overset{\lower0.5em\hbox{$\smash{\scriptscriptstyle\frown}$}}{\psi } (0)\int_{0}^{t} {\hat{\varphi }{\text{(t)d}}t}  + {{\omega }}_{{\text{d}}} \overset{\lower0.5em\hbox{$\smash{\scriptscriptstyle\frown}$}}{\varphi } (0){\text{cos}}\overset{\lower0.5em\hbox{$\smash{\scriptscriptstyle\frown}$}}{\theta } (0){\text{sin}}\overset{\lower0.5em\hbox{$\smash{\scriptscriptstyle\frown}$}}{\psi } (0)\int_{0}^{t} {{\text{d}}t}  \\     & \quad  + {{\omega }}_{{\text{d}}} \overset{\lower0.5em\hbox{$\smash{\scriptscriptstyle\frown}$}}{\theta } (0){\text{sin}}\overset{\lower0.5em\hbox{$\smash{\scriptscriptstyle\frown}$}}{\psi } (0){\text{sin}}\overset{\lower0.5em\hbox{$\smash{\scriptscriptstyle\frown}$}}{\theta } (0)\int_{0}^{t} {\hat{\varphi }{\text{(t)d}}t}  - {{\omega }}_{{\text{d}}} {\text{sin}}\overset{\lower0.5em\hbox{$\smash{\scriptscriptstyle\frown}$}}{\psi } (0){\text{sin}}\overset{\lower0.5em\hbox{$\smash{\scriptscriptstyle\frown}$}}{\theta } (0)\int_{0}^{t} {\hat{\varphi }{\text{(t)}}\hat{\theta }{\text{(t)d}}t}  \\     & \quad  + {{\omega }}_{{\text{d}}} \overset{\lower0.5em\hbox{$\smash{\scriptscriptstyle\frown}$}}{\varphi } (0){\text{sin}}\overset{\lower0.5em\hbox{$\smash{\scriptscriptstyle\frown}$}}{\psi } (0){\text{sin}}\overset{\lower0.5em\hbox{$\smash{\scriptscriptstyle\frown}$}}{\theta } (0)\int_{0}^{t} {\hat{\theta }{\text{(t)d}}t}  - {{\omega }}_{{\text{d}}} \overset{\lower0.5em\hbox{$\smash{\scriptscriptstyle\frown}$}}{\varphi } (0)\overset{\lower0.5em\hbox{$\smash{\scriptscriptstyle\frown}$}}{\theta } (0){\text{sin}}\overset{\lower0.5em\hbox{$\smash{\scriptscriptstyle\frown}$}}{\psi } (0){\text{sin}}\overset{\lower0.5em\hbox{$\smash{\scriptscriptstyle\frown}$}}{\theta } (0)\int_{0}^{t} {{\text{d}}t}  \\     & \quad  - \hat{\theta }(t){\text{sin}}\overset{\lower0.5em\hbox{$\smash{\scriptscriptstyle\frown}$}}{\psi } (0) + \overset{\lower0.5em\hbox{$\smash{\scriptscriptstyle\frown}$}}{\theta } (0){\text{sin}}\overset{\lower0.5em\hbox{$\smash{\scriptscriptstyle\frown}$}}{\psi } (0) + \hat{\varphi }(t){\text{cos}}\overset{\lower0.5em\hbox{$\smash{\scriptscriptstyle\frown}$}}{\psi } (0){\text{cos}}\overset{\lower0.5em\hbox{$\smash{\scriptscriptstyle\frown}$}}{\theta } (0) - \overset{\lower0.5em\hbox{$\smash{\scriptscriptstyle\frown}$}}{\varphi } (0){\text{cos}}\overset{\lower0.5em\hbox{$\smash{\scriptscriptstyle\frown}$}}{\psi } (0){\text{cos}}\overset{\lower0.5em\hbox{$\smash{\scriptscriptstyle\frown}$}}{\theta } (0) \\     & \quad  - \hat{\psi }(t)\overset{\lower0.5em\hbox{$\smash{\scriptscriptstyle\frown}$}}{\theta } (0){\text{cos}}\overset{\lower0.5em\hbox{$\smash{\scriptscriptstyle\frown}$}}{\psi } (0) + \hat{\psi }(t)\hat{\theta }(t){\text{cos}}\overset{\lower0.5em\hbox{$\smash{\scriptscriptstyle\frown}$}}{\psi } (0) - \overset{\lower0.5em\hbox{$\smash{\scriptscriptstyle\frown}$}}{\psi } (0)\hat{\theta }(t){\text{cos}}\overset{\lower0.5em\hbox{$\smash{\scriptscriptstyle\frown}$}}{\psi } (0) + \overset{\lower0.5em\hbox{$\smash{\scriptscriptstyle\frown}$}}{\psi } (0)\overset{\lower0.5em\hbox{$\smash{\scriptscriptstyle\frown}$}}{\theta } (0){\text{cos}}\overset{\lower0.5em\hbox{$\smash{\scriptscriptstyle\frown}$}}{\psi } (0) \\     & \quad  - \hat{\varphi }(t)\overset{\lower0.5em\hbox{$\smash{\scriptscriptstyle\frown}$}}{\psi } (0){\text{cos}}\overset{\lower0.5em\hbox{$\smash{\scriptscriptstyle\frown}$}}{\theta } (0){\text{sin}}\overset{\lower0.5em\hbox{$\smash{\scriptscriptstyle\frown}$}}{\psi } (0) + \hat{\varphi }(t)\hat{\psi }(t){\text{cos}}\overset{\lower0.5em\hbox{$\smash{\scriptscriptstyle\frown}$}}{\theta } (0){\text{sin}}\overset{\lower0.5em\hbox{$\smash{\scriptscriptstyle\frown}$}}{\psi } (0) - \overset{\lower0.5em\hbox{$\smash{\scriptscriptstyle\frown}$}}{\varphi } (0)\hat{\psi }(t){\text{cos}}\overset{\lower0.5em\hbox{$\smash{\scriptscriptstyle\frown}$}}{\theta } (0){\text{sin}}\overset{\lower0.5em\hbox{$\smash{\scriptscriptstyle\frown}$}}{\psi } (0) \\     & \quad  + \overset{\lower0.5em\hbox{$\smash{\scriptscriptstyle\frown}$}}{\varphi } (0)\overset{\lower0.5em\hbox{$\smash{\scriptscriptstyle\frown}$}}{\psi } (0){\text{cos}}\overset{\lower0.5em\hbox{$\smash{\scriptscriptstyle\frown}$}}{\theta } (0){\text{sin}}\overset{\lower0.5em\hbox{$\smash{\scriptscriptstyle\frown}$}}{\psi } (0) - \overset{\lower0.5em\hbox{$\smash{\scriptscriptstyle\frown}$}}{\varphi } (0)\overset{\lower0.5em\hbox{$\smash{\scriptscriptstyle\frown}$}}{\psi } (0){\text{cos}}\overset{\lower0.5em\hbox{$\smash{\scriptscriptstyle\frown}$}}{\theta } (0){\text{sin}}\overset{\lower0.5em\hbox{$\smash{\scriptscriptstyle\frown}$}}{\psi } (0) \\     & \quad  + \hat{\theta }(0){\text{sin}}\overset{\lower0.5em\hbox{$\smash{\scriptscriptstyle\frown}$}}{\psi } (0) - \overset{\lower0.5em\hbox{$\smash{\scriptscriptstyle\frown}$}}{\theta } (0){\text{sin}}\overset{\lower0.5em\hbox{$\smash{\scriptscriptstyle\frown}$}}{\psi } (0) - \hat{\varphi }(0){\text{cos}}\overset{\lower0.5em\hbox{$\smash{\scriptscriptstyle\frown}$}}{\psi } (0){\text{cos}}\overset{\lower0.5em\hbox{$\smash{\scriptscriptstyle\frown}$}}{\theta } (0) + \overset{\lower0.5em\hbox{$\smash{\scriptscriptstyle\frown}$}}{\varphi } (0){\text{cos}}\overset{\lower0.5em\hbox{$\smash{\scriptscriptstyle\frown}$}}{\psi } (0){\text{cos}}\overset{\lower0.5em\hbox{$\smash{\scriptscriptstyle\frown}$}}{\theta } (0) \\     & \quad  + \hat{\psi }(0)\overset{\lower0.5em\hbox{$\smash{\scriptscriptstyle\frown}$}}{\theta } (0){\text{cos}}\overset{\lower0.5em\hbox{$\smash{\scriptscriptstyle\frown}$}}{\psi } (0) - \hat{\psi }(0)\hat{\theta }(0){\text{cos}}\overset{\lower0.5em\hbox{$\smash{\scriptscriptstyle\frown}$}}{\psi } (0){\text{ + }}\overset{\lower0.5em\hbox{$\smash{\scriptscriptstyle\frown}$}}{\psi } (0)\hat{\theta }(0){\text{cos}}\overset{\lower0.5em\hbox{$\smash{\scriptscriptstyle\frown}$}}{\psi } (0) - \overset{\lower0.5em\hbox{$\smash{\scriptscriptstyle\frown}$}}{\psi } (0)\overset{\lower0.5em\hbox{$\smash{\scriptscriptstyle\frown}$}}{\theta } (0){\text{cos}}\overset{\lower0.5em\hbox{$\smash{\scriptscriptstyle\frown}$}}{\psi } (0) \\     & \quad  + \hat{\varphi }(0)\overset{\lower0.5em\hbox{$\smash{\scriptscriptstyle\frown}$}}{\psi } (0){\text{cos}}\overset{\lower0.5em\hbox{$\smash{\scriptscriptstyle\frown}$}}{\theta } (0){\text{sin}}\overset{\lower0.5em\hbox{$\smash{\scriptscriptstyle\frown}$}}{\psi } (0) - \hat{\varphi }(0)\hat{\psi }(0){\text{cos}}\overset{\lower0.5em\hbox{$\smash{\scriptscriptstyle\frown}$}}{\theta } (0){\text{sin}}\overset{\lower0.5em\hbox{$\smash{\scriptscriptstyle\frown}$}}{\psi } (0) + \overset{\lower0.5em\hbox{$\smash{\scriptscriptstyle\frown}$}}{\varphi } (0)\hat{\psi }(0){\text{cos}}\overset{\lower0.5em\hbox{$\smash{\scriptscriptstyle\frown}$}}{\theta } (0){\text{sin}}\overset{\lower0.5em\hbox{$\smash{\scriptscriptstyle\frown}$}}{\psi } (0) \\  \end{aligned}   $$

Replacing Eqs. (12) and (28) into Eq. (26) leads to:

30 $$   \begin{aligned}   \delta \omega _{{\text{e}}} t &  =  - \frac{1}{{{\text{R}}_{{\text{e}}} }}\int_{0}^{t} {v_{{\text{n}}} {\text{(}}t{\text{)d}}t}  + \omega _{{\text{d}}} \overset{\lower0.5em\hbox{$\smash{\scriptscriptstyle\frown}$}}{\varphi } (0)\overset{\lower0.5em\hbox{$\smash{\scriptscriptstyle\frown}$}}{\psi } (0){\text{cos}}\overset{\lower0.5em\hbox{$\smash{\scriptscriptstyle\frown}$}}{\theta } (0){\text{sin}}\overset{\lower0.5em\hbox{$\smash{\scriptscriptstyle\frown}$}}{\psi } (0)\int_{0}^{t} {{\text{d}}t}  \\     & \quad  - \omega _{{\text{d}}} {\text{sin}}\overset{\lower0.5em\hbox{$\smash{\scriptscriptstyle\frown}$}}{\psi } (0)\int_{0}^{t} {\hat{\theta }(t){\text{d}}t}  + \omega _{{\text{d}}} \overset{\lower0.5em\hbox{$\smash{\scriptscriptstyle\frown}$}}{\theta } (0){\text{sin}}\overset{\lower0.5em\hbox{$\smash{\scriptscriptstyle\frown}$}}{\psi } (0)\int_{0}^{t} {{\text{d}}t}  + \omega _{{\text{d}}} {\text{cos}}\overset{\lower0.5em\hbox{$\smash{\scriptscriptstyle\frown}$}}{\psi } (0){\text{cos}}\overset{\lower0.5em\hbox{$\smash{\scriptscriptstyle\frown}$}}{\theta } (0)\int_{0}^{t} {\hat{\varphi }(t){\text{d}}t}  \\     & \quad  - \omega _{{\text{d}}} \overset{\lower0.5em\hbox{$\smash{\scriptscriptstyle\frown}$}}{\varphi } (0){\text{cos}}\overset{\lower0.5em\hbox{$\smash{\scriptscriptstyle\frown}$}}{\psi } (0){\text{cos}}\overset{\lower0.5em\hbox{$\smash{\scriptscriptstyle\frown}$}}{\theta } (0)\int_{0}^{t} {{\text{d}}t}  - \omega _{{\text{d}}} \overset{\lower0.5em\hbox{$\smash{\scriptscriptstyle\frown}$}}{\theta } (0){\text{cos}}\overset{\lower0.5em\hbox{$\smash{\scriptscriptstyle\frown}$}}{\psi } (0)\int_{0}^{t} {\hat{\psi }(t){\text{d}}t}  + \omega _{{\text{d}}} {\text{cos}}\overset{\lower0.5em\hbox{$\smash{\scriptscriptstyle\frown}$}}{\psi } (0)\int_{0}^{t} {\hat{\psi }(t)\hat{\theta }(t){\text{d}}t}  \\     & \quad  - \omega _{{\text{d}}} \overset{\lower0.5em\hbox{$\smash{\scriptscriptstyle\frown}$}}{\psi } (0){\text{cos}}\overset{\lower0.5em\hbox{$\smash{\scriptscriptstyle\frown}$}}{\psi } (0)\int_{0}^{t} {\hat{\theta }(t){\text{d}}t}  + \omega _{{\text{d}}} \overset{\lower0.5em\hbox{$\smash{\scriptscriptstyle\frown}$}}{\psi } (0)\overset{\lower0.5em\hbox{$\smash{\scriptscriptstyle\frown}$}}{\theta } (0){\text{cos}}\overset{\lower0.5em\hbox{$\smash{\scriptscriptstyle\frown}$}}{\psi } (0)\int_{0}^{t} {{\text{d}}t}  - \omega _{{\text{d}}} \overset{\lower0.5em\hbox{$\smash{\scriptscriptstyle\frown}$}}{\psi } (0){\text{cos}}\overset{\lower0.5em\hbox{$\smash{\scriptscriptstyle\frown}$}}{\theta } (0){\text{sin}}\overset{\lower0.5em\hbox{$\smash{\scriptscriptstyle\frown}$}}{\psi } (0)\int_{0}^{t} {\hat{\varphi }(t){\text{d}}t}  \\     & \quad  + \omega _{{\text{d}}} {\text{cos}}\overset{\lower0.5em\hbox{$\smash{\scriptscriptstyle\frown}$}}{\theta } (0){\text{sin}}\overset{\lower0.5em\hbox{$\smash{\scriptscriptstyle\frown}$}}{\psi } (0)\int_{0}^{t} {\hat{\varphi }(t)\hat{\psi }(t){\text{d}}t}  - \omega _{{\text{d}}} \overset{\lower0.5em\hbox{$\smash{\scriptscriptstyle\frown}$}}{\varphi } (0){\text{cos}}\overset{\lower0.5em\hbox{$\smash{\scriptscriptstyle\frown}$}}{\theta } (0){\text{sin}}\overset{\lower0.5em\hbox{$\smash{\scriptscriptstyle\frown}$}}{\psi } (0)\int_{0}^{t} {\hat{\psi }(t){\text{d}}t}  \\     & \quad  + \omega _{{\text{n}}} {\text{sin}}\overset{\lower0.5em\hbox{$\smash{\scriptscriptstyle\frown}$}}{\theta } (0)\int_{0}^{t} {\hat{\varphi }(t){\text{d}}t}  - \omega _{{\text{n}}} \overset{\lower0.5em\hbox{$\smash{\scriptscriptstyle\frown}$}}{\varphi } (0){\text{sin}}\overset{\lower0.5em\hbox{$\smash{\scriptscriptstyle\frown}$}}{\theta } (0)\int_{0}^{t} {{\text{d}}t}  - \omega _{{\text{n}}} \int_{0}^{t} {\hat{\psi }(t){\text{d}}t}  + \omega _{{\text{n}}} \overset{\lower0.5em\hbox{$\smash{\scriptscriptstyle\frown}$}}{\psi } (0)\int_{0}^{t} {{\text{d}}t}  \\     & \quad  + \omega _{{\text{n}}} \overset{\lower0.5em\hbox{$\smash{\scriptscriptstyle\frown}$}}{\theta } (0){\text{cos}}^{{\text{2}}} \overset{\lower0.5em\hbox{$\smash{\scriptscriptstyle\frown}$}}{\psi } (0){\text{cos}}\overset{\lower0.5em\hbox{$\smash{\scriptscriptstyle\frown}$}}{\theta } (0)\int_{0}^{t} {\hat{\varphi }(t){\text{d}}t}  - \omega _{{\text{n}}} {\text{cos}}^{{\text{2}}} \overset{\lower0.5em\hbox{$\smash{\scriptscriptstyle\frown}$}}{\psi } (0){\text{cos}}\overset{\lower0.5em\hbox{$\smash{\scriptscriptstyle\frown}$}}{\theta } (0)\int_{0}^{t} {\hat{\varphi }(t)\hat{\theta }(t){\text{d}}t}  \\     & \quad  + \omega _{{\text{n}}} \overset{\lower0.5em\hbox{$\smash{\scriptscriptstyle\frown}$}}{\varphi } (0){\text{cos}}^{{\text{2}}} \overset{\lower0.5em\hbox{$\smash{\scriptscriptstyle\frown}$}}{\psi } (0){\text{cos}}\overset{\lower0.5em\hbox{$\smash{\scriptscriptstyle\frown}$}}{\theta } (0)\int_{0}^{t} {\hat{\theta }(t){\text{d}}t}  - \omega _{{\text{n}}} \overset{\lower0.5em\hbox{$\smash{\scriptscriptstyle\frown}$}}{\varphi } (0)\overset{\lower0.5em\hbox{$\smash{\scriptscriptstyle\frown}$}}{\theta } (0){\text{cos}}^{{\text{2}}} \overset{\lower0.5em\hbox{$\smash{\scriptscriptstyle\frown}$}}{\psi } (0){\text{cos}}\overset{\lower0.5em\hbox{$\smash{\scriptscriptstyle\frown}$}}{\theta } (0)\int_{0}^{t} {{\text{d}}t}  \\     & \quad  + \omega _{{\text{n}}} \overset{\lower0.5em\hbox{$\smash{\scriptscriptstyle\frown}$}}{\theta } (0){\text{cos}}\overset{\lower0.5em\hbox{$\smash{\scriptscriptstyle\frown}$}}{\psi } (0){\text{cos}}\overset{\lower0.5em\hbox{$\smash{\scriptscriptstyle\frown}$}}{\theta } (0){\text{sin}}\overset{\lower0.5em\hbox{$\smash{\scriptscriptstyle\frown}$}}{\psi } (0)\int_{0}^{t} {\hat{\varphi }(t)\hat{\psi }(t){\text{d}}t}  - \omega _{{\text{n}}} {\text{cos}}\overset{\lower0.5em\hbox{$\smash{\scriptscriptstyle\frown}$}}{\psi } (0){\text{cos}}\overset{\lower0.5em\hbox{$\smash{\scriptscriptstyle\frown}$}}{\theta } (0){\text{sin}}\overset{\lower0.5em\hbox{$\smash{\scriptscriptstyle\frown}$}}{\psi } (0)\int_{0}^{t} {\hat{\varphi }(t)\hat{\psi }(t)\hat{\theta }(t){\text{d}}t}  \\     & \quad  + \omega _{{\text{n}}} \overset{\lower0.5em\hbox{$\smash{\scriptscriptstyle\frown}$}}{\psi } (0){\text{cos}}\overset{\lower0.5em\hbox{$\smash{\scriptscriptstyle\frown}$}}{\psi } (0){\text{cos}}\overset{\lower0.5em\hbox{$\smash{\scriptscriptstyle\frown}$}}{\theta } (0){\text{sin}}\overset{\lower0.5em\hbox{$\smash{\scriptscriptstyle\frown}$}}{\psi } (0)\int_{0}^{t} {\hat{\varphi }(t)\hat{\theta }(t){\text{d}}t}  + \omega _{{\text{n}}} \overset{\lower0.5em\hbox{$\smash{\scriptscriptstyle\frown}$}}{\varphi } (0){\text{cos}}\overset{\lower0.5em\hbox{$\smash{\scriptscriptstyle\frown}$}}{\psi } (0){\text{cos}}\overset{\lower0.5em\hbox{$\smash{\scriptscriptstyle\frown}$}}{\theta } (0){\text{sin}}\overset{\lower0.5em\hbox{$\smash{\scriptscriptstyle\frown}$}}{\psi } (0)\int_{0}^{t} {\hat{\psi }(t)\hat{\theta }(t){\text{d}}t}  \\     & \quad  - \omega _{{\text{n}}} \overset{\lower0.5em\hbox{$\smash{\scriptscriptstyle\frown}$}}{\psi } (0)\overset{\lower0.5em\hbox{$\smash{\scriptscriptstyle\frown}$}}{\theta } (0){\text{cos}}\overset{\lower0.5em\hbox{$\smash{\scriptscriptstyle\frown}$}}{\psi } (0){\text{cos}}\overset{\lower0.5em\hbox{$\smash{\scriptscriptstyle\frown}$}}{\theta } (0){\text{sin}}\overset{\lower0.5em\hbox{$\smash{\scriptscriptstyle\frown}$}}{\psi } (0)\int_{0}^{t} {\hat{\varphi }(t){\text{d}}t}  - \omega _{{\text{n}}} \overset{\lower0.5em\hbox{$\smash{\scriptscriptstyle\frown}$}}{\varphi } (0)\overset{\lower0.5em\hbox{$\smash{\scriptscriptstyle\frown}$}}{\theta } (0){\text{cos}}\overset{\lower0.5em\hbox{$\smash{\scriptscriptstyle\frown}$}}{\psi } (0){\text{cos}}\overset{\lower0.5em\hbox{$\smash{\scriptscriptstyle\frown}$}}{\theta } (0){\text{sin}}\overset{\lower0.5em\hbox{$\smash{\scriptscriptstyle\frown}$}}{\psi } (0)\int_{0}^{t} {\hat{\psi }(t){\text{d}}t}  \\     & \quad  - \omega _{{\text{n}}} \overset{\lower0.5em\hbox{$\smash{\scriptscriptstyle\frown}$}}{\varphi } (0)\overset{\lower0.5em\hbox{$\smash{\scriptscriptstyle\frown}$}}{\psi } (0){\text{cos}}\overset{\lower0.5em\hbox{$\smash{\scriptscriptstyle\frown}$}}{\psi } (0){\text{cos}}\overset{\lower0.5em\hbox{$\smash{\scriptscriptstyle\frown}$}}{\theta } (0){\text{sin}}\overset{\lower0.5em\hbox{$\smash{\scriptscriptstyle\frown}$}}{\psi } (0)\int_{0}^{t} {\hat{\theta }(t){\text{d}}t}  + \omega _{{\text{n}}} \overset{\lower0.5em\hbox{$\smash{\scriptscriptstyle\frown}$}}{\varphi } (0)\overset{\lower0.5em\hbox{$\smash{\scriptscriptstyle\frown}$}}{\psi } (0)\overset{\lower0.5em\hbox{$\smash{\scriptscriptstyle\frown}$}}{\theta } (0){\text{cos}}\overset{\lower0.5em\hbox{$\smash{\scriptscriptstyle\frown}$}}{\psi } (0){\text{cos}}\overset{\lower0.5em\hbox{$\smash{\scriptscriptstyle\frown}$}}{\theta } (0){\text{sin}}\overset{\lower0.5em\hbox{$\smash{\scriptscriptstyle\frown}$}}{\psi } (0)\int_{0}^{t} {{\text{d}}t}  \\     & \quad  + \hat{\theta }(t){\text{cos}}\overset{\lower0.5em\hbox{$\smash{\scriptscriptstyle\frown}$}}{\psi } (0) - \overset{\lower0.5em\hbox{$\smash{\scriptscriptstyle\frown}$}}{\theta } (0){\text{cos}}\overset{\lower0.5em\hbox{$\smash{\scriptscriptstyle\frown}$}}{\psi } (0) + \hat{\varphi }(t){\text{cos}}\overset{\lower0.5em\hbox{$\smash{\scriptscriptstyle\frown}$}}{\theta } (0){\text{sin}}\overset{\lower0.5em\hbox{$\smash{\scriptscriptstyle\frown}$}}{\psi } (0) - \overset{\lower0.5em\hbox{$\smash{\scriptscriptstyle\frown}$}}{\varphi } (0){\text{cos}}\overset{\lower0.5em\hbox{$\smash{\scriptscriptstyle\frown}$}}{\theta } (0){\text{sin}}\overset{\lower0.5em\hbox{$\smash{\scriptscriptstyle\frown}$}}{\psi } (0) \\     & \quad  - \hat{\varphi }(t)\overset{\lower0.5em\hbox{$\smash{\scriptscriptstyle\frown}$}}{\theta } (0){\text{sin}}\overset{\lower0.5em\hbox{$\smash{\scriptscriptstyle\frown}$}}{\psi } (0){\text{sin}}\overset{\lower0.5em\hbox{$\smash{\scriptscriptstyle\frown}$}}{\theta } (0) + \hat{\varphi }(t)\hat{\theta }(t){\text{sin}}\overset{\lower0.5em\hbox{$\smash{\scriptscriptstyle\frown}$}}{\psi } 
(0){\text{sin}}\overset{\lower0.5em\hbox{$\smash{\scriptscriptstyle\frown}$}}{\theta } (0) - \overset{\lower0.5em\hbox{$\smash{\scriptscriptstyle\frown}$}}{\varphi } (0)\hat{\theta }(t){\text{sin}}\overset{\lower0.5em\hbox{$\smash{\scriptscriptstyle\frown}$}}{\psi } (0){\text{sin}}\overset{\lower0.5em\hbox{$\smash{\scriptscriptstyle\frown}$}}{\theta } (0) \\     & \quad  - \hat{\theta }(0){\text{cos}}\overset{\lower0.5em\hbox{$\smash{\scriptscriptstyle\frown}$}}{\psi } (0) + \overset{\lower0.5em\hbox{$\smash{\scriptscriptstyle\frown}$}}{\theta } (0){\text{cos}}\overset{\lower0.5em\hbox{$\smash{\scriptscriptstyle\frown}$}}{\psi } (0) - \hat{\varphi }(0){\text{cos}}\overset{\lower0.5em\hbox{$\smash{\scriptscriptstyle\frown}$}}{\theta } (0){\text{sin}}\overset{\lower0.5em\hbox{$\smash{\scriptscriptstyle\frown}$}}{\psi } (0) \\     & \quad  + \overset{\lower0.5em\hbox{$\smash{\scriptscriptstyle\frown}$}}{\varphi } (0){\text{cos}}\overset{\lower0.5em\hbox{$\smash{\scriptscriptstyle\frown}$}}{\theta } (0){\text{sin}}\overset{\lower0.5em\hbox{$\smash{\scriptscriptstyle\frown}$}}{\psi } (0) + \hat{\varphi }(0)\overset{\lower0.5em\hbox{$\smash{\scriptscriptstyle\frown}$}}{\theta } (0){\text{sin}}\overset{\lower0.5em\hbox{$\smash{\scriptscriptstyle\frown}$}}{\psi } (0){\text{sin}}\overset{\lower0.5em\hbox{$\smash{\scriptscriptstyle\frown}$}}{\theta } (0) - \hat{\varphi }(0)\hat{\theta }(0){\text{sin}}\overset{\lower0.5em\hbox{$\smash{\scriptscriptstyle\frown}$}}{\psi } (0){\text{sin}}\overset{\lower0.5em\hbox{$\smash{\scriptscriptstyle\frown}$}}{\theta } (0) \\     & \quad  + \overset{\lower0.5em\hbox{$\smash{\scriptscriptstyle\frown}$}}{\varphi } (0)\hat{\theta }(0){\text{sin}}\overset{\lower0.5em\hbox{$\smash{\scriptscriptstyle\frown}$}}{\psi } (0){\text{sin}}\overset{\lower0.5em\hbox{$\smash{\scriptscriptstyle\frown}$}}{\theta } (0) - \overset{\lower0.5em\hbox{$\smash{\scriptscriptstyle\frown}$}}{\varphi } (0)\overset{\lower0.5em\hbox{$\smash{\scriptscriptstyle\frown}$}}{\theta } (0){\text{sin}}\overset{\lower0.5em\hbox{$\smash{\scriptscriptstyle\frown}$}}{\psi } (0){\text{sin}}\overset{\lower0.5em\hbox{$\smash{\scriptscriptstyle\frown}$}}{\theta } (0) \\     & \quad  + \overset{\lower0.5em\hbox{$\smash{\scriptscriptstyle\frown}$}}{\varphi } (0)\overset{\lower0.5em\hbox{$\smash{\scriptscriptstyle\frown}$}}{\theta } (0){\text{sin}}\overset{\lower0.5em\hbox{$\smash{\scriptscriptstyle\frown}$}}{\psi } (0){\text{sin}}\overset{\lower0.5em\hbox{$\smash{\scriptscriptstyle\frown}$}}{\theta } (0) \\  \end{aligned}  $$

Replacing Eqs. (12) and (28) into Eq. (27) leads to:

31 $$   \begin{aligned}   \delta \omega _{{\text{d}}} t &  =  - \frac{{\tan \lambda }}{{{\text{R}}_{{\text{e}}} }}\int_{0}^{t} {v_{{\text{e}}} {\text{(}}t{\text{)d}}t}  + \omega _{{\text{n}}} \overset{\lower0.5em\hbox{$\smash{\scriptscriptstyle\frown}$}}{\varphi } (0)\overset{\lower0.5em\hbox{$\smash{\scriptscriptstyle\frown}$}}{\theta } (0){\text{sin}}\overset{\lower0.5em\hbox{$\smash{\scriptscriptstyle\frown}$}}{\psi } (0){\text{sin}}\overset{\lower0.5em\hbox{$\smash{\scriptscriptstyle\frown}$}}{\theta } (0)\int_{0}^{t} {{\text{d}}t}  \\     & \quad  + \omega _{{\text{n}}} {\text{cos}}\overset{\lower0.5em\hbox{$\smash{\scriptscriptstyle\frown}$}}{\psi } (0)\int_{0}^{t} {\hat{\theta }{\text{(t)d}}t}  - \omega _{{\text{n}}} \overset{\lower0.5em\hbox{$\smash{\scriptscriptstyle\frown}$}}{\theta } (0){\text{cos}}\overset{\lower0.5em\hbox{$\smash{\scriptscriptstyle\frown}$}}{\psi } (0)\int_{0}^{t} {{\text{d}}t}  + \omega _{{\text{n}}} {\text{cos}}\overset{\lower0.5em\hbox{$\smash{\scriptscriptstyle\frown}$}}{\theta } (0){\text{sin}}\overset{\lower0.5em\hbox{$\smash{\scriptscriptstyle\frown}$}}{\psi } (0)\int_{0}^{t} {\hat{\varphi }{\text{(t)d}}t}  \\     & \quad  - \omega _{{\text{n}}} \overset{\lower0.5em\hbox{$\smash{\scriptscriptstyle\frown}$}}{\varphi } (0){\text{cos}}\overset{\lower0.5em\hbox{$\smash{\scriptscriptstyle\frown}$}}{\theta } (0){\text{sin}}\overset{\lower0.5em\hbox{$\smash{\scriptscriptstyle\frown}$}}{\psi } (0)\int_{0}^{t} {{\text{d}}t}  - \omega _{{\text{n}}} \overset{\lower0.5em\hbox{$\smash{\scriptscriptstyle\frown}$}}{\theta } (0){\text{sin}}\overset{\lower0.5em\hbox{$\smash{\scriptscriptstyle\frown}$}}{\psi } (0){\text{sin}}\overset{\lower0.5em\hbox{$\smash{\scriptscriptstyle\frown}$}}{\theta } (0)\int_{0}^{t} {\hat{\varphi }{\text{(t)d}}t}  \\     & \quad  + \omega _{{\text{n}}} {\text{sin}}\overset{\lower0.5em\hbox{$\smash{\scriptscriptstyle\frown}$}}{\psi } (0){\text{sin}}\overset{\lower0.5em\hbox{$\smash{\scriptscriptstyle\frown}$}}{\theta } (0)\int_{0}^{t} {\hat{\varphi }{\text{(t)}}\hat{\theta }{\text{(t)d}}t}  - \omega _{{\text{n}}} \overset{\lower0.5em\hbox{$\smash{\scriptscriptstyle\frown}$}}{\varphi } (0){\text{sin}}\overset{\lower0.5em\hbox{$\smash{\scriptscriptstyle\frown}$}}{\psi } (0){\text{sin}}\overset{\lower0.5em\hbox{$\smash{\scriptscriptstyle\frown}$}}{\theta } (0)\int_{0}^{t} {\hat{\theta }{\text{(t)d}}t}  \\     & \quad  - \hat{\varphi }(t){\text{sin}}\overset{\lower0.5em\hbox{$\smash{\scriptscriptstyle\frown}$}}{\theta } (0) + \overset{\lower0.5em\hbox{$\smash{\scriptscriptstyle\frown}$}}{\varphi } (0){\text{sin}}\overset{\lower0.5em\hbox{$\smash{\scriptscriptstyle\frown}$}}{\theta } (0) + \hat{\psi }(t) - \overset{\lower0.5em\hbox{$\smash{\scriptscriptstyle\frown}$}}{\psi } (0) \\     & \quad  - \hat{\varphi }(t)\overset{\lower0.5em\hbox{$\smash{\scriptscriptstyle\frown}$}}{\theta } (0){\text{cos}}^{{\text{2}}} \overset{\lower0.5em\hbox{$\smash{\scriptscriptstyle\frown}$}}{\psi } (0){\text{cos}}\overset{\lower0.5em\hbox{$\smash{\scriptscriptstyle\frown}$}}{\theta } (0) + \hat{\varphi }(t)\hat{\theta }(t){\text{cos}}^{{\text{2}}} \overset{\lower0.5em\hbox{$\smash{\scriptscriptstyle\frown}$}}{\psi } (0){\text{cos}}\overset{\lower0.5em\hbox{$\smash{\scriptscriptstyle\frown}$}}{\theta } (0) \\     & \quad  - \overset{\lower0.5em\hbox{$\smash{\scriptscriptstyle\frown}$}}{\varphi } (0)\hat{\theta }(t){\text{cos}}^{{\text{2}}} \overset{\lower0.5em\hbox{$\smash{\scriptscriptstyle\frown}$}}{\psi } (0){\text{cos}}\overset{\lower0.5em\hbox{$\smash{\scriptscriptstyle\frown}$}}{\theta } (0) + \overset{\lower0.5em\hbox{$\smash{\scriptscriptstyle\frown}$}}{\varphi } (0)\overset{\lower0.5em\hbox{$\smash{\scriptscriptstyle\frown}$}}{\theta } (0){\text{cos}}^{{\text{2}}} \overset{\lower0.5em\hbox{$\smash{\scriptscriptstyle\frown}$}}{\psi } (0){\text{cos}}\overset{\lower0.5em\hbox{$\smash{\scriptscriptstyle\frown}$}}{\theta } (0) \\     & \quad  - \hat{\varphi }(t)\hat{\psi }(t)\overset{\lower0.5em\hbox{$\smash{\scriptscriptstyle\frown}$}}{\theta } (0){\text{cos}}\overset{\lower0.5em\hbox{$\smash{\scriptscriptstyle\frown}$}}{\psi } (0){\text{cos}}\overset{\lower0.5em\hbox{$\smash{\scriptscriptstyle\frown}$}}{\theta } (0){\text{sin}}\overset{\lower0.5em\hbox{$\smash{\scriptscriptstyle\frown}$}}{\psi } (0) + \hat{\varphi }(t)\hat{\psi }(t)\hat{\theta }(t){\text{cos}}\overset{\lower0.5em\hbox{$\smash{\scriptscriptstyle\frown}$}}{\psi } (0){\text{cos}}\overset{\lower0.5em\hbox{$\smash{\scriptscriptstyle\frown}$}}{\theta } (0){\text{sin}}\overset{\lower0.5em\hbox{$\smash{\scriptscriptstyle\frown}$}}{\psi } (0) \\     & \quad  - \hat{\varphi }(t)\overset{\lower0.5em\hbox{$\smash{\scriptscriptstyle\frown}$}}{\psi } (0)\hat{\theta }(t){\text{cos}}\overset{\lower0.5em\hbox{$\smash{\scriptscriptstyle\frown}$}}{\psi } (0){\text{cos}}\overset{\lower0.5em\hbox{$\smash{\scriptscriptstyle\frown}$}}{\theta } (0){\text{sin}}\overset{\lower0.5em\hbox{$\smash{\scriptscriptstyle\frown}$}}{\psi } (0) + \overset{\lower0.5em\hbox{$\smash{\scriptscriptstyle\frown}$}}{\varphi } (0)\hat{\psi }(t)\overset{\lower0.5em\hbox{$\smash{\scriptscriptstyle\frown}$}}{\theta } (0){\text{cos}}\overset{\lower0.5em\hbox{$\smash{\scriptscriptstyle\frown}$}}{\psi } (0){\text{cos}}\overset{\lower0.5em\hbox{$\smash{\scriptscriptstyle\frown}$}}{\theta } (0){\text{sin}}\overset{\lower0.5em\hbox{$\smash{\scriptscriptstyle\frown}$}}{\psi } (0) \\     & \quad  - \overset{\lower0.5em\hbox{$\smash{\scriptscriptstyle\frown}$}}{\varphi } (0)\hat{\psi }(t)\hat{\theta }(t){\text{cos}}\overset{\lower0.5em\hbox{$\smash{\scriptscriptstyle\frown}$}}{\psi } (0){\text{cos}}\overset{\lower0.5em\hbox{$\smash{\scriptscriptstyle\frown}$}}{\theta } (0){\text{sin}}\overset{\lower0.5em\hbox{$\smash{\scriptscriptstyle\frown}$}}{\psi } (0) + \hat{\varphi }(t)\overset{\lower0.5em\hbox{$\smash{\scriptscriptstyle\frown}$}}{\psi } (0)\overset{\lower0.5em\hbox{$\smash{\scriptscriptstyle\frown}$}}{\theta } (0){\text{cos}}\overset{\lower0.5em\hbox{$\smash{\scriptscriptstyle\frown}$}}{\psi } (0){\text{cos}}\overset{\lower0.5em\hbox{$\smash{\scriptscriptstyle\frown}$}}{\theta } (0){\text{sin}}\overset{\lower0.5em\hbox{$\smash{\scriptscriptstyle\frown}$}}{\psi } (0) \\     & \quad  + \overset{\lower0.5em\hbox{$\smash{\scriptscriptstyle\frown}$}}{\varphi } (0)\overset{\lower0.5em\hbox{$\smash{\scriptscriptstyle\frown}$}}{\psi } (0)\hat{\theta }(t){\text{cos}}\overset{\lower0.5em\hbox{$\smash{\scriptscriptstyle\frown}$}}{\psi } (0){\text{cos}}\overset{\lower0.5em\hbox{$\smash{\scriptscriptstyle\frown}$}}{\theta } (0){\text{sin}}\overset{\lower0.5em\hbox{$\smash{\scriptscriptstyle\frown}$}}{\psi } (0) - \overset{\lower0.5em\hbox{$\smash{\scriptscriptstyle\frown}$}}{\varphi } (0)\overset{\lower0.5em\hbox{$\smash{\scriptscriptstyle\frown}$}}{\psi } (0)\overset{\lower0.5em\hbox{$\smash{\scriptscriptstyle\frown}$}}{\theta } (0){\text{cos}}\overset{\lower0.5em\hbox{$\smash{\scriptscriptstyle\frown}$}}{\psi } (0){\text{cos}}\overset{\lower0.5em\hbox{$\smash{\scriptscriptstyle\frown}$}}{\theta } (0){\text{sin}}\overset{\lower0.5em\hbox{$\smash{\scriptscriptstyle\frown}$}}{\psi } (0) \\     & \quad  + \hat{\varphi }(0){\text{sin}}\overset{\lower0.5em\hbox{$\smash{\scriptscriptstyle\frown}$}}{\theta } (0) - \overset{\lower0.5em\hbox{$\smash{\scriptscriptstyle\frown}$}}{\varphi } (0){\text{sin}}\overset{\lower0.5em\hbox{$\smash{\scriptscriptstyle\frown}$}}{\theta } (0) - \hat{\psi }(0) + \overset{\lower0.5em\hbox{$\smash{\scriptscriptstyle\frown}$}}{\psi } (0) + \hat{\varphi }(0)\overset{\lower0.5em\hbox{$\smash{\scriptscriptstyle\frown}$}}{\psi } (0)\hat{\theta }(0){\text{cos}}\overset{\lower0.5em\hbox{$\smash{\scriptscriptstyle\frown}$}}{\psi } (0){\text{cos}}\overset{\lower0.5em\hbox{$\smash{\scriptscriptstyle\frown}$}}{\theta } (0){\text{sin}}\overset{\lower0.5em\hbox{$\smash{\scriptscriptstyle\frown}$}}{\psi } (0) \\     & \quad  + \hat{\varphi }(0)\overset{\lower0.5em\hbox{$\smash{\scriptscriptstyle\frown}$}}{\theta } (0){\text{cos}}^{{\text{2}}} \overset{\lower0.5em\hbox{$\smash{\scriptscriptstyle\frown}$}}{\psi } (0){\text{cos}}\overset{\lower0.5em\hbox{$\smash{\scriptscriptstyle\frown}$}}{\theta } (0) - \hat{\varphi }(0)\hat{\theta }(0){\text{cos}}^{{\text{2}}} \overset{\lower0.5em\hbox{$\smash{\scriptscriptstyle\frown}$}}{\psi } (0){\text{cos}}\overset{\lower0.5em\hbox{$\smash{\scriptscriptstyle\frown}$}}{\theta } (0) \\     & \quad  + \overset{\lower0.5em\hbox{$\smash{\scriptscriptstyle\frown}$}}{\varphi } (0)\hat{\theta }(0){\text{cos}}^{{\text{2}}} \overset{\lower0.5em\hbox{$\smash{\scriptscriptstyle\frown}$}}{\psi } (0){\text{cos}}\overset{\lower0.5em\hbox{$\smash{\scriptscriptstyle\frown}$}}{\theta } (0) - \overset{\lower0.5em\hbox{$\smash{\scriptscriptstyle\frown}$}}{\varphi } (0)\overset{\lower0.5em\hbox{$\smash{\scriptscriptstyle\frown}$}}{\theta } (0){\text{cos}}^{{\text{2}}} \overset{\lower0.5em\hbox{$\smash{\scriptscriptstyle\frown}$}}{\psi } (0){\text{cos}}\overset{\lower0.5em\hbox{$\smash{\scriptscriptstyle\frown}$}}{\theta } (0) \\     & \quad  + \hat{\varphi }(0)\hat{\psi }(0)\overset{\lower0.5em\hbox{$\smash{\scriptscriptstyle\frown}$}}{\theta } (0){\text{cos}}\overset{\lower0.5em\hbox{$\smash{\scriptscriptstyle\frown}$}}{\psi } (0){\text{cos}}\overset{\lower0.5em\hbox{$\smash{\scriptscriptstyle\frown}$}}{\theta } (0){\text{sin}}\overset{\lower0.5em\hbox{$\smash{\scriptscriptstyle\frown}$}}{\psi } (0) - \hat{\varphi }(0)\hat{\psi }(0)\hat{\theta }(0){\text{cos}}\overset{\lower0.5em\hbox{$\smash{\scriptscriptstyle\frown}$}}{\psi } (0){\text{cos}}\overset{\lower0.5em\hbox{$\smash{\scriptscriptstyle\frown}$}}{\theta } (0){\text{sin}}\overset{\lower0.5em\hbox{$\smash{\scriptscriptstyle\frown}$}}{\psi } (0) \\     & \quad  + \overset{\lower0.5em\hbox{$\smash{\scriptscriptstyle\frown}$}}{\varphi } (0)\hat{\psi }(0)\hat{\theta }(0){\text{cos}}\overset{\lower0.5em\hbox{$\smash{\scriptscriptstyle\frown}$}}{\psi } 
(0){\text{cos}}\overset{\lower0.5em\hbox{$\smash{\scriptscriptstyle\frown}$}}{\theta } (0){\text{sin}}\overset{\lower0.5em\hbox{$\smash{\scriptscriptstyle\frown}$}}{\psi } (0) - \hat{\varphi }(0)\overset{\lower0.5em\hbox{$\smash{\scriptscriptstyle\frown}$}}{\psi } (0)\overset{\lower0.5em\hbox{$\smash{\scriptscriptstyle\frown}$}}{\theta } (0){\text{cos}}\overset{\lower0.5em\hbox{$\smash{\scriptscriptstyle\frown}$}}{\psi } (0){\text{cos}}\overset{\lower0.5em\hbox{$\smash{\scriptscriptstyle\frown}$}}{\theta } (0){\text{sin}}\overset{\lower0.5em\hbox{$\smash{\scriptscriptstyle\frown}$}}{\psi } (0) \\     & \quad  - \overset{\lower0.5em\hbox{$\smash{\scriptscriptstyle\frown}$}}{\varphi } (0)\overset{\lower0.5em\hbox{$\smash{\scriptscriptstyle\frown}$}}{\psi } (0)\hat{\theta }(0){\text{cos}}\overset{\lower0.5em\hbox{$\smash{\scriptscriptstyle\frown}$}}{\psi } (0){\text{cos}}\overset{\lower0.5em\hbox{$\smash{\scriptscriptstyle\frown}$}}{\theta } (0){\text{sin}}\overset{\lower0.5em\hbox{$\smash{\scriptscriptstyle\frown}$}}{\psi } (0) + \overset{\lower0.5em\hbox{$\smash{\scriptscriptstyle\frown}$}}{\varphi } (0)\overset{\lower0.5em\hbox{$\smash{\scriptscriptstyle\frown}$}}{\psi } (0)\overset{\lower0.5em\hbox{$\smash{\scriptscriptstyle\frown}$}}{\theta } (0){\text{cos}}\overset{\lower0.5em\hbox{$\smash{\scriptscriptstyle\frown}$}}{\psi } (0){\text{cos}}\overset{\lower0.5em\hbox{$\smash{\scriptscriptstyle\frown}$}}{\theta } (0){\text{sin}}\overset{\lower0.5em\hbox{$\smash{\scriptscriptstyle\frown}$}}{\psi } (0) \\     & \quad  - \overset{\lower0.5em\hbox{$\smash{\scriptscriptstyle\frown}$}}{\varphi } (0)\hat{\psi }(0)\overset{\lower0.5em\hbox{$\smash{\scriptscriptstyle\frown}$}}{\theta } (0){\text{cos}}\overset{\lower0.5em\hbox{$\smash{\scriptscriptstyle\frown}$}}{\psi } (0){\text{cos}}\overset{\lower0.5em\hbox{$\smash{\scriptscriptstyle\frown}$}}{\theta } (0){\text{sin}}\overset{\lower0.5em\hbox{$\smash{\scriptscriptstyle\frown}$}}{\psi } (0) \\  \end{aligned}  $$

According to Eqs. (29)–(31), the projection of gyros error in the navigation coordinate are obtained; these errors can be compensated, and thus the IMU accuracy is improved. In the next section, the extracted equations are simulated and verified.

## Methods

**Figure 1:**
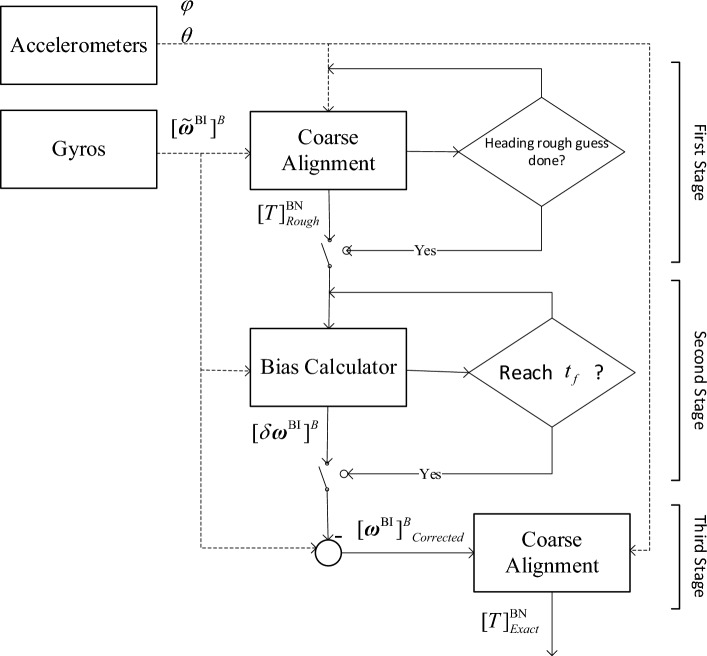
Flowchart of the proposed alignment algorithm.

The objective of this section is to provide a detailed explanation of a novel algorithm that aligns SINS systems fast and precisely. The main innovation of this algorithm lies in its use of analytical relations to identify bias errors of gyroscopes and then eliminate them from IMU outputs. Unlike traditional initial alignment algorithms that involve a laborious fine alignment phase, the proposed approach can estimate initial Euler angles by substituting the fine alignment phase with a conventional coarse alignment process. This results in a substantial reduction in the time required to achieve a high level of accuracy. In other words, the proposed algorithm gains the fast computation time of inaccurate conventional coarse alignment phase and accuracy of traditional time-consuming fine alignment techniques at the same time. Figure 1 illustrates a general view of the suggested alignment algorithm. It should be mentioned that the proposed algorithm is operational in the stationary alignment condition.

This algorithm consists of three stages, with the first and third stages being single-shot processes, while the second stage is an iterative process that has a maximum iteration time of T_f_. The first stage involves a conventional coarse alignment algorithm while the second stage is the primary contribution of this paper and calculates bias errors in accelerometers and gyroscopes using analytical relations derived in the previous section. Once the second stage reaches its maximum running time condition, the third stage begins by subtracting calculated biases from gyros and accelerometers output and then running a conventional coarse alignment, similar to the first stage. The proposed alignment requires a time period which equals the time required by the coarse alignment and achieves an accuracy which is given by the fine alignment. The algorithm utilizes analytical explicit formulas and there is no tuning procedure as in the fine alignment.

In the proposed algorithm, at first, the average value of the IMU’s outputs are computed. This averaging is done to remove the noise effects and causes six numbers (three for accelerometers and three for gyros). The following procedure is then performed:

1. Coarse alignment is executed (by the six numbers from the averaged IMU’s outputs).
2. Differential calibration is done (by the six numbers from the averaged IMU’s outputs and three numbers from the coarse alignment output as the initial condition for roll, pitch and yaw).
3. Another coarse alignment is performed (using the six numbers from the averaged IMU’s outputs and six numbers from the differential calibration output as the biases of accelerometers and gyros).

In above procedure, calibration of inertial sensors (step 2) is performed to remove biases from the six averaged numbers; The averaged numbers are used in steps 1, 2 and 3. In steps 1 and 2, the averaged numbers are contaminated to biases. In step 3, biases are removed from the averaged numbers.

## Simulation and experiments

In this section, the relations extracted in the previous section are simulated and verified. The error model of accelerometers and gyroscopes, including misalignment error, scale factor, bias, and noise are assumed as the following:

32 $$ \left[ {\begin{array}{*{20}c} {\tilde{f}_{{\text{x}}} } \\ {\tilde{f}_{{\text{y}}} } \\ {\tilde{f}_{{\text{z}}} } \\ \end{array} } \right] = \left[ {\begin{array}{*{20}c} {S_{{\text{x}}}^{{\text{a}}} } & {M_{{{\text{xy}}}}^{{\text{a}}} } & {M_{{{\text{xz}}}}^{{\text{a}}} } \\ {M_{{{\text{yx}}}}^{{\text{a}}} } & {S_{{\text{y}}}^{{\text{a}}} } & {M_{{{\text{yz}}}}^{{\text{a}}} } \\ {M_{{{\text{zx}}}}^{{\text{a}}} } & {M_{{{\text{zy}}}}^{{\text{a}}} } & {S_{{\text{z}}}^{{\text{a}}} } \\ \end{array} } \right]\left[ {\begin{array}{*{20}c} {f_{{\text{x}}} } \\ {f_{{\text{y}}} } \\ {f_{{\text{z}}} } \\ \end{array} } \right] + \left[ {\begin{array}{*{20}c} {b_{{\text{x}}}^{{\text{a}}} } \\ {b_{{\text{y}}}^{{\text{a}}} } \\ {b_{{\text{z}}}^{{\text{a}}} } \\ \end{array} } \right] + \left[ {\begin{array}{*{20}c} {n_{{\text{x}}}^{{\text{a}}} } \\ {n_{{\text{y}}}^{{\text{a}}} } \\ {n_{{\text{z}}}^{{\text{a}}} } \\ \end{array} } \right] $$

33 $$ \left[ {\begin{array}{*{20}c} {\tilde{\omega }_{{\text{x}}} } \\ {\tilde{\omega }_{{\text{y}}} } \\ {\tilde{\omega }_{{\text{z}}} } \\ \end{array} } \right] = \left[ {\begin{array}{*{20}c} {S_{{\text{x}}}^{{\text{g}}} } & {M_{{{\text{xy}}}}^{{\text{g}}} } & {M_{{{\text{xz}}}}^{{\text{g}}} } \\ {M_{{{\text{yx}}}}^{{\text{g}}} } & {S_{{\text{y}}}^{{\text{g}}} } & {M_{{{\text{yz}}}}^{{\text{g}}} } \\ {M_{{{\text{zx}}}}^{{\text{g}}} } & {M_{{{\text{zy}}}}^{{\text{g}}} } & {S_{{\text{z}}}^{{\text{g}}} } \\ \end{array} } \right]\left[ {\begin{array}{*{20}c} {\omega_{{\text{x}}} } \\ {\omega_{{\text{y}}} } \\ {\omega_{{\text{z}}} } \\ \end{array} } \right] + \left[ {\begin{array}{*{20}c} {b_{{\text{x}}}^{{\text{g}}} } \\ {b_{{\text{y}}}^{{\text{g}}} } \\ {b_{{\text{z}}}^{{\text{g}}} } \\ \end{array} } \right] + \left[ {\begin{array}{*{20}c} {n_{{\text{x}}}^{{\text{g}}} } \\ {n_{{\text{y}}}^{{\text{g}}} } \\ {n_{{\text{z}}}^{{\text{g}}} } \\ \end{array} } \right] $$

To verify Eqs. (23), (29)–(31), various scenarios have been created according to Tables 1, 2 and 3. In Table 1, 50 different conditions for the inertial block are generated randomly (including latitude, longitude, altitude, and Euler angles). The calibration error coefficient for accelerometers and gyros are generated in Tables 2 and 3, respectively. Based on Tables 1, 2 and 3, 50 different scenarios are produced.

**Table 1:** Definition of position and attitude in different scenarios.

Sc no.	λ (deg)	ℓ (deg)	h (m)	φ (deg)	θ (deg)	ψ (deg)
1	52.063	29.042	1965	46.613	73.551	143.531
2	52.703	303.545	4451	− 62.945	− 71.000	− 39.908
3	7.125	308.086	3704	− 55.853	77.477	6.554
4	− 77.400	288.496	2940	119.151	− 74.700	− 32.002
5	40.374	313.443	62	− 27.888	− 34.659	39.528
6	31.124	267.927	4982	− 54.630	− 30.206	44.526
7	52.991	253.733	3046	172.458	− 73.953	162.428
8	31.898	233.046	92	− 120.062	61.330	− 137.091
9	34.875	227.766	3193	169.242	83.826	− 89.233
10	24.498	202.075	4250	− 11.065	− 64.584	79.098
11	− 32.001	103.863	3017	60.075	− 24.025	− 21.306
12	− 65.192	107.609	1364	− 133.940	− 6.788	110.531
13	− 62.814	350.914	3815	− 177.923	11.451	112.853
14	− 30.661	293.175	2503	85.684	56.627	79.929
15	− 25.950	300.372	4870	10.787	− 38.505	18.294
16	27.598	165.953	2164	75.236	− 79.447	− 143.084
17	32.153	41.740	2898	26.452	− 33.117	− 160.984
18	− 20.918	263.982	3393	109.971	8.385	− 32.586
19	− 39.757	202.430	3966	120.247	72.776	− 72.402
20	− 8.421	163.970	1029	− 138.234	18.998	− 165.802
21	− 12.792	345.385	1775	− 7.166	− 38.086	62.434
22	− 51.257	21.917	3929	− 50.191	76.107	− 67.129
23	− 66.248	288.103	930	− 82.876	− 82.749	83.469
24	25.062	316.380	2370	− 125.611	66.468	− 95.655
25	− 77.872	115.560	4338	− 138.943	67.078	64.595
26	48.196	265.293	2489	18.614	74.229	23.576
27	19.453	69.024	3860	− 177.341	− 72.813	− 93.902
28	4.776	304.305	2128	1.206	− 39.348	− 75.370
29	− 8.743	62.418	4259	28.027	65.844	− 136.211
30	59.229	30.576	4280	61.957	58.603	− 173.401
31	− 23.558	61.844	4762	70.082	− 46.028	115.519
32	− 12.654	38.284	82	− 108.154	78.009	− 3.288
33	32.204	125.333	3072	− 159.569	− 85.415	94.240
34	35.560	35.200	2417	143.509	− 71.756	53.949
35	− 44.924	75.912	524	− 37.448	− 70.225	100.154
36	81.921	80.399	1864	103.743	− 9.152	− 126.512
37	− 25.089	319.960	2407	33.837	68.994	− 113.941
38	76.504	187.387	495	33.956	73.464	150.176
39	18.113	328.424	3865	8.183	− 49.128	70.935
40	− 44.545	294.023	4809	− 20.745	83.960	− 120.771
41	− 57.278	163.223	4066	− 3.620	9.313	− 173.292
42	− 8.749	233.549	4672	125.186	− 79.628	129.391
43	79.156	47.619	83	− 106.696	− 74.997	151.264
44	5.280	291.591	3031	78.776	3.58	− 10.292
45	82.904	289.473	401	− 142.101	18.234	148.797
46	34.143	59.349	2270	109.561	46.448	120.330
47	− 42.880	271.319	619	− 35.522	− 37.395	− 113.109
48	50.011	251.843	3230	8.939	− 63.217	54.644
49	74.671	120.871	547	− 80.063	72.806	47.665
50	− 75.426	166.792	3366	31.516	− 61.217	− 139.371

**Table 2:** Definition of accelerometers coefficients in different scenarios.

Sc No.	$S_{{\text{x}}}^{{\text{a}}}$	$M_{{{\text{xy}}}}^{{\text{a}}}$	$M_{{{\text{xz}}}}^{{\text{a}}}$	$M_{{{\text{yx}}}}^{{\text{a}}}$	$S_{{\text{y}}}^{{\text{a}}}$	$M_{{{\text{yz}}}}^{{\text{a}}}$	$M_{{{\text{zx}}}}^{{\text{a}}}$	$M_{{{\text{zy}}}}^{{\text{a}}}$	$S_{{\text{z}}}^{{\text{a}}}$	$b_{{\text{x}}}^{{\text{a}}}$	$b_{{\text{y}}}^{{\text{a}}}$	$b_{{\text{z}}}^{{\text{a}}}$
	ppm	s	s	s	ppm	s	s	s	ppm	μg	μg	μg
1	− 49	6	12	6	− 37	14	12	9	− 50	55	123	147
2	− 27	14	7	6	− 58	15	9	13	− 58	133	148	85
3	− 67	13	8	14	− 73	11	15	14	− 45	119	83	130
4	− 49	10	7	10	− 34	14	7	6	− 52	72	106	100
5	− 41	9	13	13	− 39	13	8	5	− 57	73	99	140
6	− 67	9	8	7	− 43	9	7	6	− 43	99	134	108
7	− 34	11	13	10	− 26	12	5	6	− 55	67	141	57
8	− 49	12	6	12	− 43	7	8	7	− 56	82	100	120
9	− 33	9	13	10	− 32	6	11	11	− 48	141	139	125
10	− 55	5	8	9	− 72	6	8	6	− 30	114	140	63
11	− 51	5	8	9	− 42	12	7	14	− 37	142	144	90
12	− 41	12	10	7	− 49	13	11	15	− 63	71	85	136
13	− 35	11	6	5	− 61	13	7	13	− 27	112	55	129
14	− 69	7	12	7	− 69	10	12	14	− 52	90	69	109
15	− 69	6	13	11	− 35	11	10	10	− 71	112	74	125
16	− 48	6	10	11	− 57	8	7	12	− 56	75	82	86
17	− 28	7	13	7	− 73	11	7	10	− 72	52	115	112
18	− 73	8	13	8	− 31	6	7	9	− 54	119	94	130
19	− 36	7	11	13	− 33	13	14	8	− 45	136	106	130
20	− 54	5	12	6	− 26	8	9	10	− 74	82	96	115
21	− 41	12	10	14	− 51	8	12	12	− 48	68	89	81
22	− 67	12	12	7	− 49	5	14	9	− 70	93	72	120
23	− 56	14	9	14	− 28	8	11	8	− 34	138	70	94
24	− 44	15	13	15	− 41	13	8	9	− 34	95	73	108
25	− 71	12	11	13	− 32	13	14	6	− 59	149	124	92
26	− 61	7	10	8	− 51	6	11	13	− 29	86	110	70
27	− 36	11	10	8	− 66	10	10	14	− 38	76	103	66
28	− 74	14	7	6	− 65	15	7	12	− 64	138	146	58
29	− 38	8	13	12	− 70	6	9	6	− 33	54	109	72
30	− 55	11	10	10	− 74	8	6	14	− 39	109	121	72
31	− 36	9	11	9	− 44	12	5	15	− 47	72	137	107
32	− 41	14	8	13	− 51	9	15	11	− 50	85	124	133
33	− 50	10	8	13	− 50	11	5	8	− 56	128	140	71
34	− 40	13	15	6	− 67	9	6	7	− 30	125	106	140
35	− 53	9	10	12	− 37	5	13	7	− 73	140	110	99
36	− 46	14	9	6	− 75	5	11	6	− 48	82	112	83
37	− 41	8	10	14	− 54	15	13	8	− 32	123	86	58
38	− 53	12	15	11	− 35	10	11	13	− 62	89	79	99
39	− 44	5	8	7	− 66	11	6	6	− 60	106	93	70
40	− 31	15	13	6	− 51	14	10	14	− 71	79	54	83
41	− 58	11	8	7	− 40	14	13	7	− 51	134	61	118
42	− 37	6	7	13	− 40	11	12	8	− 59	95	68	92
43	− 64	10	11	10	− 33	10	11	6	− 57	122	118	91
44	− 70	15	11	10	− 25	6	10	10	− 46	143	111	102
45	− 26	11	14	12	− 71	9	15	7	− 62	120	83	58
46	− 64	10	13	6	− 60	14	13	9	− 70	146	117	108
47	− 58	13	8	14	− 36	13	7	11	− 34	143	86	134
48	− 46	13	10	5	− 61	14	11	11	− 32	71	82	55
49	− 36	6	10	14	− 63	11	9	11	− 71	144	75	106
50	− 50	15	5	11	− 69	6	7	8	− 65	104	116	93

**Table 3:** Definition of gyros coefficients in different scenarios.

Sc No.	$S_{{\text{x}}}^{{\text{g}}}$	$M_{{{\text{xy}}}}^{{\text{g}}}$	$M_{{{\text{xz}}}}^{{\text{g}}}$	$M_{{{\text{yx}}}}^{{\text{g}}}$	$S_{{\text{y}}}^{{\text{g}}}$	$M_{{{\text{yz}}}}^{{\text{g}}}$	$M_{{{\text{zx}}}}^{{\text{g}}}$	$M_{{{\text{zy}}}}^{{\text{g}}}$	$S_{{\text{z}}}^{{\text{g}}}$	$b_{{\text{x}}}^{{\text{g}}}$	$b_{{\text{y}}}^{{\text{g}}}$	$b_{{\text{z}}}^{{\text{g}}}$
	ppm	s	s	s	ppm	s	s	s	ppm	°/h	°/h	°/h
1	− 22	6	5	6	− 22	6	4	3	− 16	0.012	0.009	0.005
2	− 25	7	3	6	− 13	5	4	7	− 30	0.014	0.008	0.005
3	− 13	4	3	3	− 10	3	5	7	− 25	0.005	0.006	0.008
4	− 22	3	6	6	− 15	3	7	7	− 11	0.015	0.010	0.006
5	− 29	6	5	7	− 18	3	5	7	− 27	0.014	0.005	0.008
6	− 10	4	7	5	− 12	3	3	6	− 11	0.011	0.009	0.014
7	− 13	5	3	3	− 20	6	3	4	− 11	0.006	0.005	0.012
8	− 29	3	5	5	− 27	6	6	7	− 25	0.014	0.009	0.011
9	− 15	3	6	6	− 19	3	5	6	− 15	0.015	0.011	0.008
10	− 19	6	3	3	− 13	6	6	4	− 13	0.007	0.006	0.014
11	− 11	3	7	4	− 24	4	5	7	− 28	0.010	0.010	0.010
12	− 25	4	5	6	− 16	6	3	4	− 25	0.009	0.006	0.010
13	− 29	7	5	7	− 14	6	7	3	− 29	0.007	0.015	0.008
14	− 14	5	7	6	− 22	3	7	5	− 29	0.012	0.009	0.007
15	− 22	6	6	6	− 19	6	4	7	− 11	0.014	0.015	0.014
16	− 25	7	4	7	− 10	5	7	3	− 13	0.012	0.014	0.009
17	− 19	7	3	3	− 28	6	5	7	− 23	0.014	0.013	0.006
18	− 23	5	6	3	− 13	6	4	3	− 25	0.015	0.009	0.014
19	− 15	7	3	6	− 30	5	7	3	− 12	0.012	0.013	0.008
20	− 26	6	6	5	− 11	6	7	7	− 14	0.010	0.014	0.014
21	− 28	6	4	3	− 24	4	7	6	− 19	0.010	0.006	0.009
22	− 24	5	3	5	− 27	3	5	7	− 26	0.015	0.013	0.012
23	− 26	7	5	4	− 28	7	6	4	− 16	0.009	0.009	0.010
24	− 27	5	3	3	− 26	6	7	5	− 23	0.008	0.011	0.010
25	− 20	3	7	7	− 22	6	6	7	− 30	0.013	0.008	0.007
26	− 26	7	6	5	− 26	3	6	4	− 28	0.011	0.014	0.010
27	− 20	5	6	6	− 15	4	5	7	− 17	0.008	0.010	0.012
28	− 13	5	4	7	− 17	5	4	4	− 29	0.011	0.015	0.008
29	− 13	3	5	6	− 23	6	7	5	− 16	0.008	0.007	0.009
30	− 20	4	6	3	− 22	7	4	5	− 17	0.010	0.006	0.008
31	− 29	5	3	4	− 20	4	6	7	− 25	0.005	0.008	0.009
32	− 19	5	3	3	− 22	5	7	5	− 29	0.009	0.012	0.011
33	− 20	4	4	7	− 29	3	6	6	− 12	0.006	0.008	0.007
34	− 19	6	7	5	− 18	3	7	7	− 14	0.009	0.006	0.013
35	− 20	3	4	3	− 28	4	3	5	− 23	0.014	0.012	0.008
36	− 11	5	3	6	− 23	4	6	4	− 16	0.009	0.006	0.006
37	− 11	4	7	6	− 12	7	4	6	− 18	0.012	0.005	0.013
38	− 16	3	7	6	− 12	3	7	7	− 25	0.009	0.015	0.014
39	− 29	4	3	6	− 26	6	7	4	− 12	0.010	0.011	0.006
40	− 18	7	5	5	− 25	4	3	6	− 16	0.013	0.006	0.008
41	− 29	6	3	7	− 26	5	6	5	− 23	0.015	0.013	0.005
42	− 11	5	7	3	− 10	3	3	7	− 15	0.011	0.006	0.010
43	− 24	7	6	6	− 21	7	3	5	− 27	0.006	0.011	0.009
44	− 14	5	3	4	− 21	4	5	3	− 29	0.015	0.013	0.007
45	− 16	3	7	6	− 16	4	7	4	− 26	0.009	0.012	0.012
46	− 16	4	4	4	− 24	3	6	6	− 13	0.010	0.005	0.015
47	− 28	3	5	4	− 14	7	5	6	− 20	0.005	0.014	0.013
48	− 22	4	4	3	− 10	3	6	4	− 24	0.013	0.013	0.005
49	− 13	3	5	6	− 20	7	4	4	− 12	0.012	0.010	0.015
50	− 24	3	6	3	− 26	5	5	5	− 13	0.008	0.007	0.006

**Figure 2:**
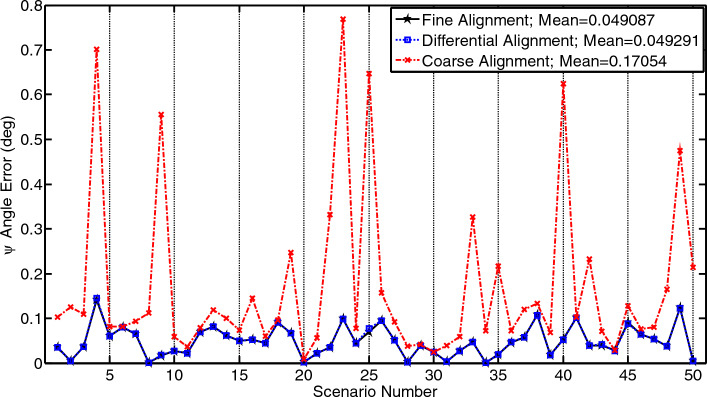
Comparison between different alignment algorithms.

**Figure 3:**
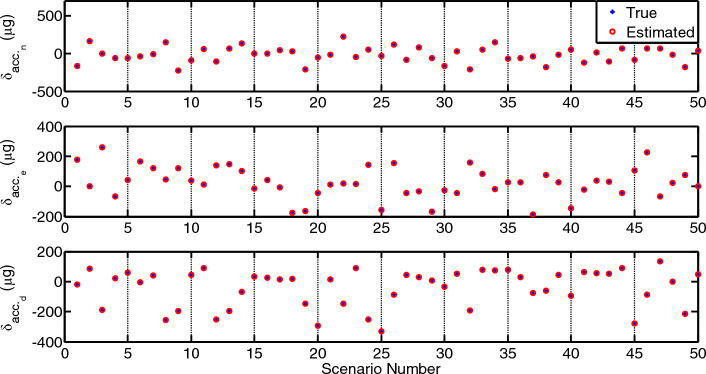
Estimated value of accelerometers error with the alignment algorithm.

**Figure 4:**
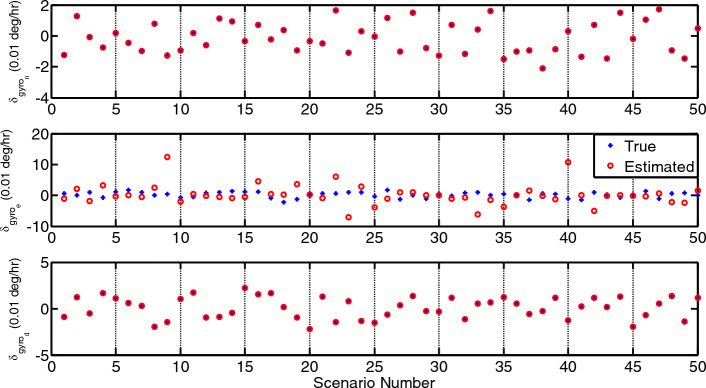
Estimated value of gyroscopes error with the alignment algorithm.

In Fig. 2, the results of the proposed algorithm are compared with the ‘coarse’ and ‘fine’ alignment methods. For this purpose, a coarse alignment is run at first. Then, the proposed algorithm is run, and the IMU error is compensated. Lastly, another coarse alignment is run by the error-compensated IMU. Results show that the proposed algorithm has achieved fine alignment accuracy in a limited time. In Fig. 3, the values of the accelerometer errors calculated by the proposed algorithm are shown. It is observed that the accelerometer errors are calculated accurately. In Fig. 4, the error values of gyros are shown. It is observed that the proposed algorithm cannot estimate the gyroscope error of the east channel, but in the north and down channels, the errors of the gyros are estimated accurately. It should be mentioned that according to Ref.^31^, east gyro error is not observable and thus not estimable.

In the subsequent tests, the FOG100 gyrocompass data produced by GEM Elettronica, Italy, was utilized to validate the performance of the proposed alignment technique in real conditions. This system consists of three optical fiber gyroscopes with an accuracy of 0.02 deg/h and three quartz accelerometers with an accuracy of 50 µg. The data sampling frequency was 100 Hz. maximum iteration time, T_f_, was set by try and error to T_f_ = 0.05 s. The device was placed on a rate table, and stationary outputs were recorded. This procedure was repeated ten times, and in each trial, five points were selected randomly (fifty points in total). The table was also used to calculate the bias of the sensors by subtracting the measured values from the nominal values.

**Figure 5:**
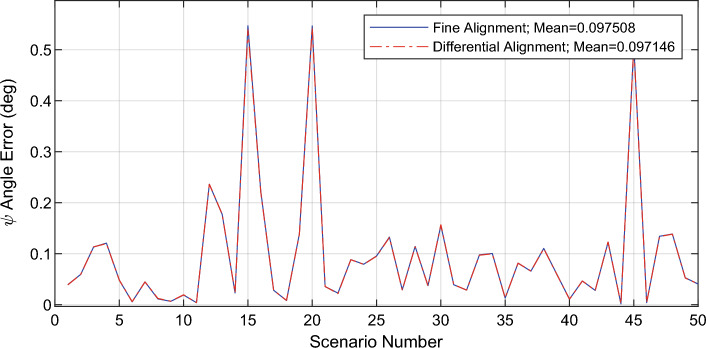
Comparison between different alignment algorithms.

**Figure 6:**
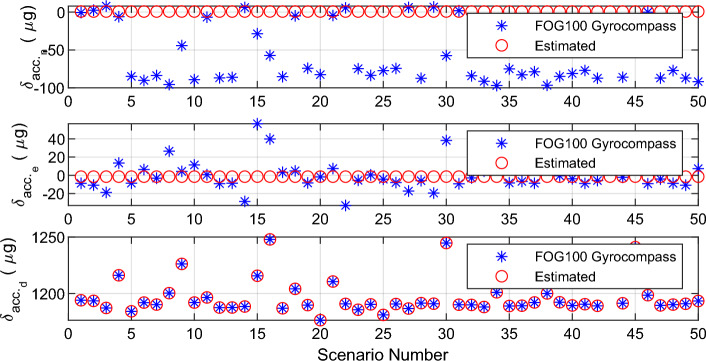
Estimated value of accelerometers error with the alignment algorithm.

**Figure 7:**
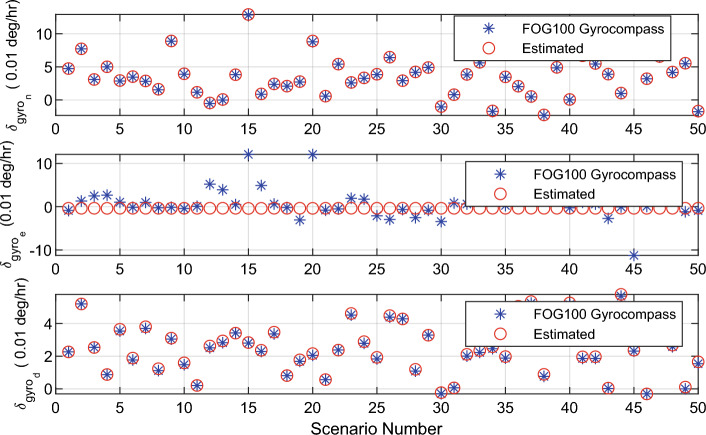
Estimated value of gyroscopes error with the alignment algorithm.

Figure 5 indicates that the proposed alignment error in gyrocompassing is very similar to the fine alignment approach. According to Appendix B, the accuracy of the proposed algorithm is the same as the fine alignment algorithm. In this figure, the errors represent the difference between the table orientation and the alignment techniques' output. Figure 6 depicts the bias of the accelerometers in the navigation frame, as well as their estimates by the proposed alignment method. According to Ref.^31^, the bias of the vertical accelerometer is observable; therefore, this bias is precisely calculated. The bias of the gyroscopes in the navigation frame is seen in Fig. 7. The north and vertical gyros biases are observable and thus they are accurately determined.

## Conclusion

This paper proposes a novel algorithm for inertial gyrocompassing in the stationary stand-alone mode. The proposed algorithm only uses an IMU to solve the initial alignment problem quickly, accurately, and reasonably. The new algorithm is fast as the ‘coarse alignment’ and accurate as the ‘fine alignment’ algorithms. For this purpose, the mathematical relation between the ‘small rotation angles’ and the ‘navigation error equations’ was extracted and used to calculate the error of IMU sensors. These errors can be compensated, and consequently, the accuracy of the IMU is improved; This, in turn, causes the initial alignment procedure to be performed more accurately. Simulations and experiments show that the proposed algorithm can achieve the accuracy of fine alignment in a short time (the same time as needed by the coarse alignment). Therefore, the proposed algorithm is superior to the two standard initial alignment algorithms (coarse and fine alignment).

### Supplementary Information

Supplementary Information.

## Data Availability

The datasets used and/or analyzed during the current study available from the corresponding author on reasonable request.

## List of symbols

$${\text{B}}$$: Body frame
$${\text{N}}$$: Navigation frame
${\hat{\text{N}}}$: Estimated navigation frame
${\text{R}}_{{\text{e}}}$: Radius of earth
$${\mathbf{E}}$$: Identity tensor
$f_{{\text{x}}} ,\,f_{{\text{y}}} ,\,f_{{\text{z}}}$: Accelerometers output in x, y, and z channels of the body frame
${\updelta }f_{{\text{x}}} ,\,{\updelta }f_{{\text{y}}} ,\,{\updelta }f_{{\text{z}}}$: The error of accelerometers in the x, y, and z channels of the body frame
${\mathbf{R}}^{{{\hat{\text{N}}\text{N}}}}$: Rotation tensor of the frame ${\hat{\text{N}}}$ relative to frame N
$v_{{\text{n}}} ,\,v_{{\text{e}}} ,\,v_{{\text{d}}}$: Velocities in north, east, and down channels
$\lambda ,\,\ell ,\,h$: Latitude, longitude, and height
${\upomega }_{{\text{n}}} ,\,{\upomega }_{{\text{d}}}$: Components of Earth's angular velocity in the north and down channels
${\upvarepsilon }$: ‘Component perturbation’ operator
${\updelta }$: ‘Total perturbation’ operator
$\omega_{{\text{x}}} ,\,\omega_{{\text{y}}} ,\,\omega_{{\text{z}}}$: Gyros output in x, y, and z channels of the body frame
${\updelta }\omega_{{\text{x}}} ,\,{\updelta }\omega_{{\text{y}}} ,\,{\updelta }\omega_{{\text{z}}}$: The error of gyros in the x, y, and z channels of the body frame
${\upvarepsilon }{\mathbf{r}}^{{{\hat{\text{N}}\text{N}}}}$: Perturbation of the rotation vector of the frame ${\hat{\text{N}}}$ relative to frame N
${\upvarepsilon }{\mathbf{R}}^{{{\hat{\text{N}}\text{N}}}}$: Skew symmetric form of ${\upvarepsilon }{\mathbf{r}}^{{{\hat{\text{N}}\text{N}}}}$
$\phi ,\,\theta ,\,\psi$: Roll, pitch, and yaw angles
$\hat{\phi },\,\hat{\theta },\,\hat{\psi }$: Estimated roll, pitch, and yaw angles
$$\overset{\lower0.5em\hbox{$\smash{\scriptscriptstyle\frown}$}}{\varphi } ,\,\overset{\lower0.5em\hbox{$\smash{\scriptscriptstyle\frown}$}}{\theta } ,\,\overset{\lower0.5em\hbox{$\smash{\scriptscriptstyle\frown}$}}{\psi }$$: Actual roll, pitch, and yaw angles
${\upvarepsilon }\phi ,\,{\upvarepsilon }\theta ,\,{\upvarepsilon }\psi$: Small rotation angles
$\Delta \phi ,\,\Delta \theta ,\,\Delta \psi$: The error in roll, pitch, and yaw angles
$[{\text{T}}]^{{{\text{BN}}}}$: Transformation matrix of the navigation frame to the body frame
$$[{\hat{\text{T}}}]^{{{\text{BN}}}}$$: Estimation of $$[{\text{T}}]^{{{\text{BN}}}}$$
