# Supplementary material for: A fast strapdown gyrocompassing algorithm based on INS differential errors
Source: Sci Rep. 2023 Sep 15;13:15297. doi: 10.1038/s41598-023-42235-6 (PMC10504366; doi:10.1038/s41598-023-42235-6)
Supplement: Supplementary file 1 — Supplementary Information. [file 41598_2023_42235_MOESM1_ESM.docx]

**Appendix A: Perturbation Operators**

The Component perturbation operator, introduced in [24], is used to obtain a tensorial form of attitude error equations. This operator is defined as:

| $\text{ε}\mathbf{x}=\hat{\mathbf{x}}-\mathbf{R}^{\hat{\text{D}}\text{D}}\mathbf{x}$ | (34) |
| --- | --- |

where $\mathbf{R}^{\hat{\text{D}}\text{D}}$ represents the rotation tensor of the perturbed frame $\hat{\text{D}}$ with respect to the reference frame $D$; $\hat{\mathbf{x}}$ is the perturbed tensor and $\mathbf{x}$ is the reference tensor. The $\varepsilon-$perturbation operator differs from the $\delta-$perturbation operator, which is defined as:

| $\text{δ}\mathbf{x}=\hat{\mathbf{x}}-\mathbf{x}$ | (35) |
| --- | --- |

If $\mathbf{x}$ is a scalar variable, the $\delta-$perturbation operator will be simplified to the $\Delta-$perturbation operator as follows:

| $\Delta\mathbf{x}=\hat{\mathbf{x}}-\mathbf{x}$ | (36) |
| --- | --- |

**Appendix B: Error Analysis**

In this appendix, the error analysis of the proposed alignment algorithm is presented. Consider an IMU in the stationary condition, accelerometers measure the surface reaction force, and gyroscopes measure the Earth’s angular velocity, both in the body frame:

| $[\tilde{\mathbf{f}}]^{\text{B}}=[-\mathbf{g}]^{\text{B}}=\left[\begin{matrix} \tilde{f}_{\text{x}} \\ \tilde{f}_{\text{y}} \\ \tilde{f}_{\text{z}} \end{matrix}\right] , [\tilde{\mathbf{\omega}}^{\text{BI}}]^{\text{B}}=[\mathbf{\omega}^{\text{EI}}]^{\text{B}}=\left[\begin{matrix} \tilde{\omega}_{\text{x}} \\ \tilde{\omega}_{\text{y}} \\ \tilde{\omega}_{\text{z}} \end{matrix}\right]$ | (37) |
| --- | --- |

The sign indicates the measurement of the desired quantity. In stationary condition, we also have:

| $[-\mathbf{g}]^{\text{B}}=[\text{T}]^{\text{BN}}[-\mathbf{g}]^{\text{N}} , [\mathbf{\omega}^{\text{EI}}]^{\text{B}}=[\text{T}]^{\text{BN}}[\mathbf{\omega}^{\text{EI}}]^{\text{N}}$ | (38) |
| --- | --- |

Considering equation (38), the following matrix can be formed:

| $\left[[-\mathbf{g}]^{\text{B}} [\mathbf{\omega}^{\text{EI}}]^{\text{B}} [-\mathbf{g}]^{\text{B}}\mathop{\times}\limits[\mathbf{\omega}^{\text{EI}}]^{\text{B}}\right]=[\text{T}]^{\text{BN}}\left[[-\mathbf{g}]^{\text{N}} [\mathbf{\omega}^{\text{EI}}]^{\text{N}} [-\mathbf{g}]^{\text{N}}\mathop{\times}\limits[\mathbf{\omega}^{\text{EI}}]^{\text{N}}\right]$ | (39) |
| --- | --- |

Now consider $\mathbf{M}=\left[[-\mathbf{g}]^{\text{N}} [\mathbf{\omega}^{\text{EI}}]^{\text{N}} [-\mathbf{g}]^{\text{N}}\mathop{\times}\limits[\mathbf{\omega}^{\text{EI}}]^{\text{N}}\right]$, we would have:

| $[\text{T}]^{\text{BN}}=\left[[-\mathbf{g}]^{\text{B}} [\mathbf{\omega}^{\text{EI}}]^{\text{B}} [-\mathbf{g}]^{\text{B}}\mathop{\times}\limits[\mathbf{\omega}^{\text{EI}}]^{\text{B}}\right] \mathbf{M}^{-1}$ | (40) |
| --- | --- |

By solving equation (40), an initial coarse estimate of the system attitude, $[\text{T}]^{\text{BN}}$, is obtained as follows:

| $[\text{T}]^{\text{BN}}\approx\left[\begin{matrix} \frac{\sec\lambda}{\text{ω}^{\text{EI}}}\tilde{\omega}_{\text{x}}-\frac{\tan\lambda}{\text{g}}\tilde{f}_{\text{x}} & \frac{\sec\lambda}{\text{gω}^{\text{EI}}}\text{(}\tilde{f}_{\text{z}}\tilde{\omega}_{\text{y}}-\tilde{f}_{\text{y}}\tilde{\omega}_{\text{z}}\text{)} & \frac{-1}{\text{g}}\tilde{f}_{\text{x}} \\ \frac{\sec\lambda}{\text{ω}^{\text{EI}}}\tilde{\omega}_{\text{y}}-\frac{\tan\lambda}{\text{g}}\tilde{f}_{\text{y}} & \frac{\sec\lambda}{\text{gω}^{\text{EI}}}\text{(}\tilde{f}_{\text{x}}\tilde{\omega}_{\text{z}}-\tilde{f}_{\text{z}}\tilde{\omega}_{\text{x}}\text{)} & \frac{-1}{\text{g}}\tilde{f}_{\text{y}} \\ \frac{\sec\lambda}{\text{ω}^{\text{EI}}}\tilde{\omega}_{\text{z}}-\frac{\tan\lambda}{\text{g}}\tilde{f}_{\text{z}} & \frac{\sec\lambda}{\text{gω}^{\text{EI}}}\text{(}\tilde{f}_{\text{y}}\tilde{\omega}_{\text{x}}-\tilde{f}_{\text{x}}\tilde{\omega}_{\text{y}}\text{)} & \frac{-1}{\text{g}}\tilde{f}_{\text{z}} \end{matrix}\right]$ | (41) |
| --- | --- |

If the output of the sensors had no error, equation (41) would also have no error and consequently the roll, pitch and yaw angles would be calculated exactly. In the following, the effect of the sensors error in the calculation of attitude matrix and Euler’s angles is investigated. According to relations (37), (38) and (40), we will have:

| $[\text{T}]^{\text{B}\hat{\text{N}}}=\left[[\tilde{\mathbf{f}}]^{\text{B}} [\tilde{\mathbf{\omega}}^{\text{BI}}]^{\text{B}} [\tilde{\mathbf{f}}]^{\text{B}}\times[\tilde{\mathbf{\omega}}^{\text{BI}}]^{\text{B}}\right] \mathbf{M}^{-1}$ | (42) |
| --- | --- |

In equation (41), $[\mathbf{T}]^{\text{B}\hat{\text{N}}}$, $[\tilde{\mathbf{f}}]^{\text{B}}$ and $[\tilde{\mathbf{\omega}}^{\text{BI}}]^{\text{B}}$ are respectively the estimation of the transfer matrix, the specific force and the angular rate sensed by the inertial sensors and are modeled as follows:

|  | (43) |
| --- | --- |

In equation (43), is the skew-symmetric form of the tilt tensor (), which shows the tilt of the calculated transfer matrix () from its real value ($[\text{T}]^{\text{NB}}$). According to equation (43), we would have:

|  | (44) |
| --- | --- |

Subtracting equation (40) from equation (44), will result in:

| $[\text{T}]^{\text{BN}}[\text{ε}\mathbf{R}^{\hat{\text{N}}\text{N}}]^{\text{N}}=\left[[\text{δ}\mathbf{f}]^{\text{B}} [\text{δ}\mathbf{\omega}^{\text{BI}}]^{\text{B}} [\text{δ}\mathbf{f}]^{\text{B}}\times[\mathbf{\omega}^{\text{EI}}]^{\text{B}}-[\mathbf{g}]^{\text{B}}\times[\text{δ}\mathbf{\omega}^{\text{BI}}]^{\text{B}}\right] \mathbf{M}^{-1}$ | (45) |
| --- | --- |

Equation (45) relates the state estimation error to the sensors error and could be rewritten as follows:

| $[\text{ε}\mathbf{R}^{\hat{\text{N}}\text{N}}]^{\text{N}}=\left[[\text{δ}\mathbf{f}]^{\text{N}} [\text{δ}\mathbf{\omega}^{\text{BI}}]^{\text{N}} [\text{δ}\mathbf{f}]^{\text{N}}\times[\mathbf{\omega}^{\text{EI}}]^{\text{N}}-[\mathbf{g}]^{\text{N}}\times[\text{δ}\mathbf{\omega}^{\text{BI}}]^{\text{N}}\right] \mathbf{M}^{-1}$ | (46) |
| --- | --- |

Now consider the following definitions:

| $[\text{δ}\mathbf{f}]^{\text{N}}=\left[\begin{matrix} \text{δ}f_{\text{n}} \\ \text{δ}f_{\text{e}} \\ \text{δ}f_{\text{d}} \end{matrix}\right] , [\text{δ}\mathbf{\omega}^{\text{BI}}]^{\text{N}}=\left[\begin{matrix} \text{δ}\omega_{\text{n}} \\ \text{δ}\omega_{\text{e}} \\ \text{δ}\omega_{\text{d}} \end{matrix}\right]$ | (47) |
| --- | --- |

Substituting equations (47) into equation (46), we would have:

| $[\text{ε}\mathbf{R}^{\hat{\text{N}}\text{N}}]^{\text{N}}=\left[\begin{matrix} +\frac{\sec\lambda}{\text{ω}^{\text{EI}}}\text{δ}\omega_{\text{d}} & -\frac{\sec\lambda}{\text{ω}^{\text{EI}}}\text{δ}\omega_{\text{e}}+\frac{\tan\lambda}{\text{g}}\text{δ}f_{\text{e}} & -\frac{1}{\text{g}}\text{δ}f_{\text{n}} \\ -\frac{\tan\lambda}{\text{g}}\text{δ}f_{\text{e}}+\frac{\sec\lambda}{\text{ω}^{\text{EI}}}\text{δ}\omega_{\text{e}} & +\frac{\sec\lambda}{\text{ω}^{\text{EI}}}\text{δ}\omega_{\text{n}}-\frac{1}{\text{g}}\text{δ}f_{\text{d}} & -\frac{1}{\text{g}}\text{δ}f_{\text{e}} \\ +\frac{1}{\text{g}}\text{δ}f_{\text{n}} & +\frac{1}{\text{g}}\text{δ}f_{\text{e}} & -\frac{1}{\text{g}}\text{δ}f_{\text{d}} \end{matrix}\right]$ | (48) |
| --- | --- |

Equation (48) shows that in the presence of sensors error, $[\text{ε}\mathbf{R}^{\hat{\text{N}}\text{N}}]^{\text{N}}$ is not necessarily skew-symmetric; But ideally, we should have:

|  | (49) |
| --- | --- |

According to the results of the proposed algorithm, it is seen that $\text{δ}f_{\text{d}}$, $\text{δ}\omega_{\text{n}}$ and $\text{δ}\omega_{\text{d}}$ are observable and $\text{δ}f_{\text{n}}$, $\text{δ}f_{\text{e}}$ and $\text{δ}\omega_{\text{e}}$ are unobservable states; It should be mentioned that the same result is obtained by performing the observability analysis to the fine alignment algorithm. Observable states are computed and compensated; thus, we would have:

| $\text{δ}f_{\text{d}}=0 , \text{δ}\omega_{\text{n}}=0 , \text{δ}\omega_{\text{d}}=0$ | (50) |
| --- | --- |

Applying equation (50) to equation (480), results in:

| $[\text{ε}\mathbf{R}^{\hat{\text{N}}\text{N}}]^{\text{N}}=\left[\begin{matrix} 0 & -\frac{\sec\lambda}{\text{ω}^{\text{EI}}}\text{δ}\omega_{\text{e}}+\frac{\tan\lambda}{\text{g}}\text{δ}f_{\text{e}} & -\frac{1}{\text{g}}\text{δ}f_{\text{n}} \\ +\frac{\sec\lambda}{\text{ω}^{\text{EI}}}\text{δ}\omega_{\text{e}}-\frac{\tan\lambda}{\text{g}}\text{δ}f_{\text{e}} & 0 & -\frac{1}{\text{g}}\text{δ}f_{\text{e}} \\ +\frac{1}{\text{g}}\text{δ}f_{\text{n}} & +\frac{1}{\text{g}}\text{δ}f_{\text{e}} & 0 \end{matrix}\right]$ | (51) |
| --- | --- |

Comparing equation (51) with equation (49), the error of the proposed algorithm is obtained:

|  | (52) |
| --- | --- |

The results obtained in equation (52) are in full compliance with the error analysis of the fine alignment algorithm given in [31].
